# The Optimal Type and Dose of Exercise Interventions on VEGF Levels in Healthy Individuals, as Well as Obesity and Chronic Disease Populations: A Network Meta-Analysis

**DOI:** 10.3390/biomedicines13102548

**Published:** 2025-10-19

**Authors:** Liqun Jiang, Huimin Ding, Dongjun Lee, Buongo Chun

**Affiliations:** Graduate School of Physical Education, Myongji University, Yongin 17058, Republic of Korea; jiangliqun@mju.ac.kr (L.J.); dinghuimin@mju.ac.kr (H.D.); ldj0719@mju.ac.kr (D.L.)

**Keywords:** vascular endothelial growth factor (VEGF), exercise intervention, network meta-analysis, dose–response relationship, angiogenesis, resistance training, obesity, cardiovascular disease

## Abstract

**Background/Objectives**: Impaired angiogenesis and vascular dysfunction are central features of chronic diseases such as cardiovascular disorders, neurodegeneration, and metabolic syndrome. Vascular endothelial growth factor (VEGF) plays a pivotal role in vascular repair and metabolic regulation, yet its responses to exercise remain inconsistently reported. This study aimed to systematically compare the effects of different exercise modalities and doses on VEGF levels across diverse populations. **Methods**: This review was registered in PROSPERO (CRD42025643709) and followed PRISMA guidelines. PubMed, Web of Science, Embase, and Cochrane Library were searched until 16 January 2025. Eligible studies were randomized or quasi-experimental trials reporting exercise-induced changes in serum/plasma VEGF. Data were extracted and assessed independently using JBI tools. Exercise types were categorized and doses standardized as metabolic equivalents (METs). Network meta-analysis was performed in Stata17.0 (SMD as effect size), with SUCRA used for ranking. Dose–response relationships were examined by meta-regression (remr package), and publication bias was assessed via funnel plots. **Results**: Twenty-eight studies (N = 1138) were included. In healthy adults, lower-limb resistance training produced the greatest VEGF increase, with benefits observed above ~600 METs-min/week and peaking near 1950 METs-min/week. Among obese individuals, combined aerobic and resistance training under hypoxic conditions showed the highest VEGF response, though dose-specific effects were not significant. In patients with chronic conditions, upper-limb resistance training within 756–950 METs-min/week was most effective, displaying a U-shaped dose–response relationship. No substantial publication bias was detected. **Conclusions**: The VEGF response to exercise appears to be influenced by both population characteristics and training dosage. High-intensity lower-limb resistance training may provide greater benefits for healthy adults, while obese individuals might experience enhanced responses with combined training under hypoxic conditions. For clinical populations, moderate-dose upper-limb resistance training may be particularly beneficial. Large-scale, long-term trials are needed to further clarify and refine exercise prescriptions targeting VEGF-mediated vascular adaptations.

## 1. Introduction

Chronic diseases such as cardiovascular disorders, neurodegenerative conditions, and metabolic syndrome remain the leading global causes of morbidity and mortality, with impaired angiogenesis and vascular dysfunction as shared pathological hallmarks [1,2]. These processes not only restrict tissue perfusion and repair but also accelerate disease progression and functional decline [3,4,5]. Identifying safe and effective interventions to restore vascular function and metabolic homeostasis has therefore become a central focus of both basic and clinical research.

Against this backdrop, vascular endothelial growth factor (VEGF) has emerged as a key signaling molecule regulating angiogenesis and vascular repair [6]. Dysregulated VEGF expression has been closely linked to multiple diseases: in idiopathic pulmonary fibrosis, insufficient angiogenesis accelerates structural remodeling and loss of lung function [7,8]; in early Alzheimer’s disease, reduced cerebrospinal fluid VEGF levels correlate with cognitive decline [9,10]; in ischemic heart disease, inadequate VEGF expression exacerbates myocardial hypoperfusion and increases the risk of ischemic cardiomyopathy [11]; and in obesity, adipose tissue overexpression of VEGF improves insulin sensitivity and alleviates inflammation [12]. Collectively, these findings highlight VEGF as indispensable for vascular homeostasis, neuroprotection, and metabolic regulation. Nevertheless, excessive VEGF expression may be deleterious, contributing to increased vascular permeability, cerebral edema, and tumor progression, underscoring its “double-edged sword” nature [13].

Among the diverse strategies proposed to modulate VEGF, exercise has attracted particular interest due to its safety, low cost, and systemic benefits. Increasing evidence suggests that exercise can transiently or moderately upregulate VEGF through hypoxia-driven pathways, thereby enhancing angiogenesis and vascular function. For example, acute resistance exercise (e.g., leg extensions) markedly increased VEGF mRNA and protein expression in skeletal muscle, with circulating VEGF levels peaking 2–4 h post-exercise [14]. Similarly, a 6-month randomized controlled trial (RCT) demonstrated that aerobic exercise significantly elevated circulating VEGF concentrations compared with controls [15]. By contrast, an RCT in sedentary postmenopausal women (50–74 years) found no significant changes in VEGF after 150 vs. 300 min of aerobic training per week, suggesting that conventional aerobic exercise doses may not strongly affect VEGF [16]. Other studies have also reported inconsistent findings: in obese postmenopausal women, 12 weeks of moderate-intensity walking failed to increase VEGF levels [17]; in patients with cardiovascular disease, however, exercise-induced mobilization of endothelial progenitor cells (EPCs), likely mediated by VEGF, was markedly greater than in healthy individuals, indicating that populations with metabolic abnormalities may be more responsive to VEGF-modulating exercise interventions [18]. Moreover, VEGF responses appear to vary widely depending on exercise intensity, duration, and frequency. Importantly, most available evidence comes from small-scale RCTs or observational studies, with dispersed results and inconsistent conclusions. High-quality evidence is still lacking to address critical questions such as which exercise modality is most effective, the optimal exercise dose, and whether differential responses exist across populations. These limitations severely hinder the clinical translation of exercise prescriptions for VEGF modulation and chronic disease prevention.

To address these evidence gaps, more systematic approaches are urgently needed. Traditional meta-analyses are generally restricted to pairwise comparisons and are therefore unable to comprehensively compare multiple exercise modalities [19] or to delineate dose–response relationships between exercise and VEGF. In this study, we therefore conducted a systematic review and network meta-analysis (NMA) to integrate the available evidence, comprehensively compare the relative efficacy of different exercise modalities on VEGF regulation [20,21], and further perform dose–response modeling to quantify the relationship between exercise volume and VEGF levels. By doing so, we aim to identify the optimal exercise strategies for VEGF modulation and provide robust evidence to guide personalized exercise prescriptions, ultimately advancing the role of exercise interventions in the prevention and management of cardiovascular, metabolic, and neurodegenerative diseases.

## 2. Methods

This dose–response network meta-analysis was prospectively registered in PROSPERO (CRD42025643709) and is reported in compliance with the PRISMA guidelines [22].

### 2.1. Search Strategy

A comprehensive literature search was conducted in PubMed, Web of Science, Embase, and the Cochrane Library from database inception to 16 January 2025. The detailed search strategies, including keywords, search dates, and procedures, are provided in Supplemental Table S1. In addition, the reference lists of eligible articles and relevant reviews were manually screened to identify additional studies. Title/abstract and full-text screening were performed independently by two reviewers, with discrepancies resolved through discussion or consultation with a third reviewer.

### 2.2. Study Selection

Study eligibility was determined according to the PICOS framework. Participants included healthy individuals or patients with non-neoplastic chronic diseases; individuals with cancer or acute conditions that could affect VEGF expression or exercise responses were excluded. Interventions comprised structured exercise programs (e.g., aerobic, resistance, high-intensity interval training [HIIT], flexibility, or combined modalities) with clearly reported type, intensity, frequency, and duration. Comparators included usual care, no intervention, or alternative exercise modalities. Outcomes required serum or plasma VEGF levels with extractable quantitative data. Eligible study designs were RCTs or quasi-experimental studies. Exclusion criteria were duplicate or inaccessible publications, incomplete outcome data, non-original articles (e.g., reviews, editorials), and trials without quantifiable differences between intervention and comparator groups.

### 2.3. Data Extraction

Two researchers independently screened titles, abstracts, and full texts, with any discrepancies resolved through discussion or consultation with a third reviewer. A standardized data extraction form was used to collect study information, including participant characteristics, intervention details, sample size, outcomes, VEGF measurement source (serum or plasma), assay method, sampling timing, and risk of bias domains. When necessary, study authors were contacted to obtain missing information, and Origin software was applied to extract numerical data from published figures.

### 2.4. Data Coding and Management

Exercise interventions were categorized into specific types, such as Low-intensity Blood Flow Restriction Resistance Training (LBFRT), Aerobic Exercise with Resistance (AEWR), and High-intensity Aerobic Exercise (HAE), among others. Exercise dose was standardized as weekly metabolic equivalents (METs-min/week), calculated as the product of exercise intensity (in METs), session duration (minutes), and frequency per week. When exercise workload was not directly reported, values were estimated using compendium-based MET scores for the corresponding activities, and for resistance training, ACSM guidelines were applied to assign approximate MET values according to relative intensity (e.g., percentage of 1RM) and workload characteristics [23,24].

### 2.5. Quality Assessment

The methodological quality of the included studies was assessed using the standardized critical appraisal tools developed by the Joanna Briggs Institute (JBI). For RCTs, the JBI checklist comprises 13 items that evaluate aspects such as randomization, adequacy of control groups, blinding, and completeness of follow-up. For quasi-experimental studies, the JBI checklist includes 9 items addressing the clarity of causal relationships, appropriateness of control groups, comparability of participants, and consistency of interventions. Two reviewers independently appraised each study, with each item rated as “Yes” or “No” to ensure objectivity and consistency in quality assessment [25,26]. In addition, the overall certainty of evidence for each outcome was evaluated using the Grading of Recommendations, Assessment, Development and Evaluation (GRADE) approach. Five domains were considered: risk of bias, inconsistency, indirectness, imprecision, and publication bias. Based on these criteria, the certainty of evidence was classified as high, moderate, low, or very low, providing a transparent framework to interpret the robustness of the pooled findings [27].

### 2.6. Statistical Analyses

Prior to analysis, all multi-arm trials were split into two-arm comparisons [28]. A network plot was generated using the “gemtc” package in R version 4.3.3 (R Foundation for Statistical Computing, Vienna, Austria) to visualize study connections and sample sizes [29]. Network meta-analysis was then conducted in Stata 17.0 (StataCorp LLC, College Station, TX, USA), with continuous outcomes converted to standardized mean differences (SMD). Global inconsistency was assessed using an inconsistency model (*p* < 0.05 indicating inconsistency), and node-splitting was applied when closed loops were present to test for local inconsistency [30]. Based on these results, either a consistency or an inconsistency model was selected. Interventions were ranked using the surface under the cumulative ranking curve (SUCRA), and results were displayed using forest plots and league tables. If the 95% confidence interval (CI) crossed 0, the effect was not significant; otherwise, a significant positive or negative effect was inferred [31]. Funnel plots were used to assess publication bias. For dose–response analysis, meta-regression was conducted in Stata using the “remr” package. VEGF change was set as the outcome and weekly exercise dose as the predictor. The model generated SMD estimates with 95% CI and *p*-values [32]. To explore sources of heterogeneity, exploratory meta-regression analyses examined whether the proportion of male participants, mean age, VEGF source (serum vs. plasma), and assay method (e.g., ELISA vs. multiplex platforms) moderated the observed VEGF effects across trials [33].

## 3. Results

### 3.1. Characteristics of Included Studies

**Figure 1:**
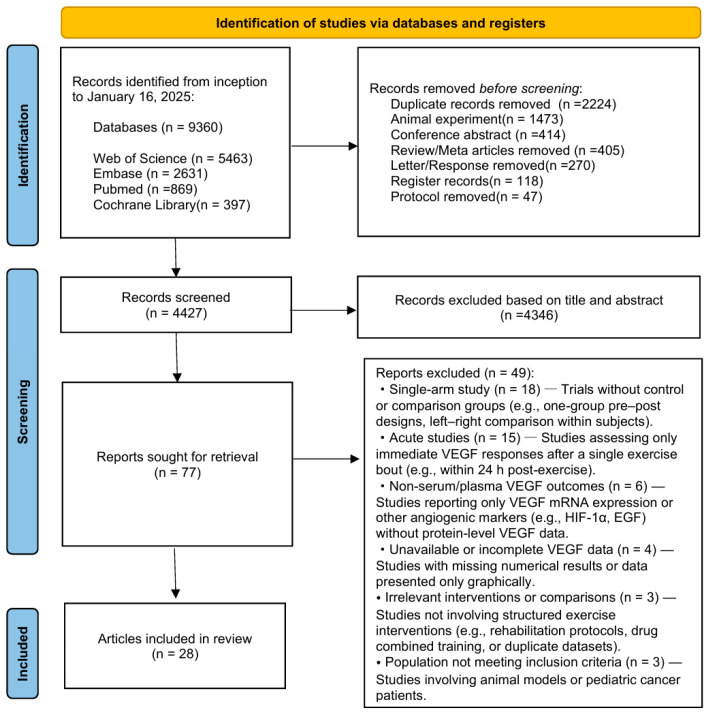
Literature screening flow chart.

A total of 9360 records were identified through systematic database searches. After excluding duplicates, conference abstracts, and animal studies, 4427 unique records were retained for screening. Following title and abstract review, 77 full-text articles were assessed, and 28 studies met the inclusion criteria (Figure 1). These included 27 randomized controlled trials and one quasi-experimental study, involving 1138 participants across 14 countries.

Study populations comprised healthy individuals (n = 11), individuals with obesity (n = 5), and patients with chronic conditions such as type 2 diabetes, hypertension, Parkinson’s disease, and Alzheimer’s disease (n = 12). The studies were published between 2008 and 2024 and exhibited diverse designs, including one five-arm, two four-arm, six three-arm, and nineteen two-arm trials. Sample sizes ranged from 16 to 189, with intervention durations of 4 weeks to 6 months, frequencies of 2–6 sessions per week, session lengths of 9–155 min, and total weekly exercise doses ranging from 200 to 3300 METs-min/week Across the 28 studies, a total of 17 distinct exercise modalities were evaluated, and all studies reported changes in vascular endothelial growth factor (VEGF) levels.

VEGF was measured using both serum (n = 14) and plasma (n = 14) samples, with assay platforms including conventional ELISA kits, multiplex ELISA, and electrochemiluminescence assays. Blood sampling was most commonly performed in the morning under fasting conditions, though a few trials collected immediate post-exercise or delayed post-intervention samples. A summary of population groups, exercise doses, VEGF assays, and main findings is provided in Table 1, while detailed study characteristics are presented in Supplemental Table S2.

### 3.2. Risk of Bias Assessment

The methodological quality of the 28 included studies was evaluated using the JBI critical appraisal tools. Overall, most trials were rated as having acceptable quality, with the majority of items judged as “Yes.” The most common methodological concerns were related to allocation concealment and blinding of participants or outcome assessors, which were frequently rated as “Unclear.” No study was judged to be at high risk of bias, and key domains such as randomization, completeness of outcome data, and baseline comparability were generally well reported. Detailed assessments are presented in Supplemental Table S3.

### 3.3. Certainty of Evidence (CINeMA Assessment)

The certainty of evidence for network comparisons was evaluated using the GRADE framework (Supplementary Tables S4.1–S4.3). In the healthy population, most comparisons were rated as low or very low, primarily downgraded for imprecision due to small sample sizes and wide confidence intervals, with only a few contrasts (e.g., AEWR vs. CG, CG vs. HAE) reaching moderate or low certainty. For obese and clinical populations, nearly all comparisons were graded as low or very low, reflecting consistent downgrading for imprecision, heterogeneity, and indirectness. Overall, the confidence in network estimates ranged from very low to moderate, underscoring the limited strength of the current evidence base.

### 3.4. Network Meta-Analysis Results

#### 3.4.1. Healthy Population

**Figure 2:**
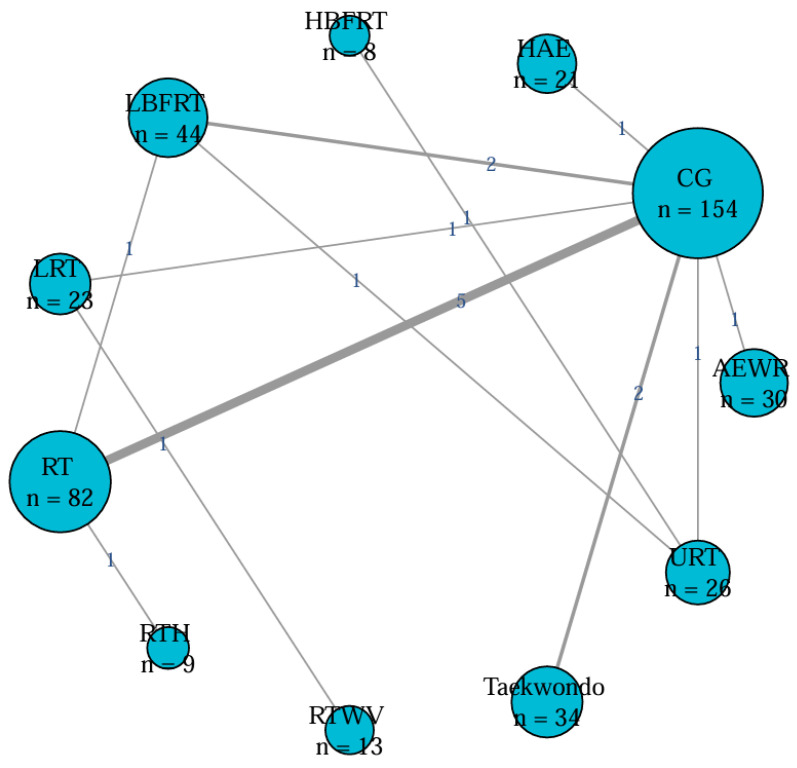
Network plot for healthy population.

**Figure 3:**
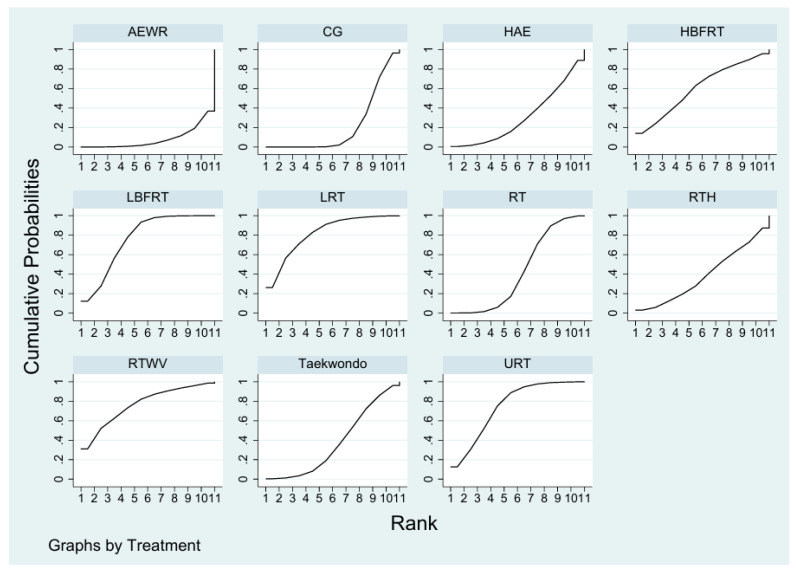
Overall SUCRA rankings for healthy population.

**Figure 4:**
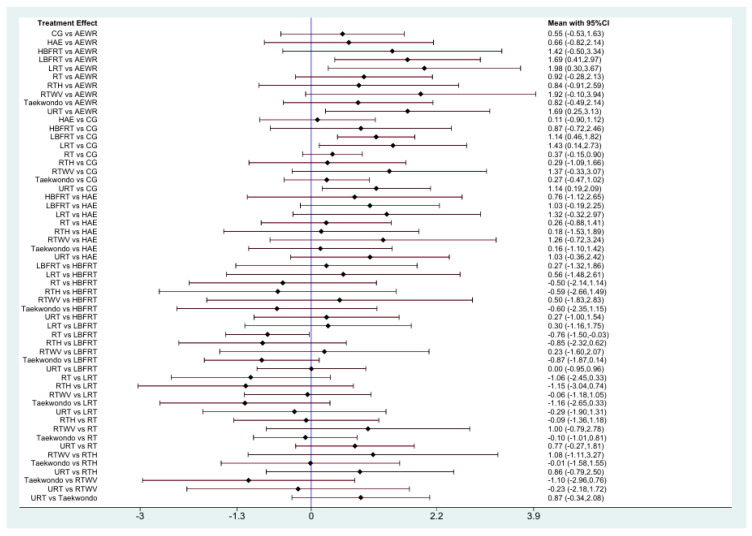
Forest plots of healthy population.

A total of 11 randomized controlled trials investigated the effects of exercise interventions on VEGF levels in healthy individuals (Figure 2). No evidence of global inconsistency was observed (*p* = 0.797), and node-splitting analysis also indicated no significant local inconsistency (all *p* > 0.05; e.g., AEWR vs. CG, *p* = 0.989) (Supplementary S5 and Table S6). SUCRA rankings (Figure 3) suggested that Lower Limb Resistance Training (LRT, SUCRA = 81.9) and Low-intensity Blood Flow Restriction Resistance Training (LBFRT, SUCRA = 76.5) were most likely to be effective, whereas Aerobic Exercise With Resistance (AEWR, SUCRA = 8.1) ranked lowest. However, several rankings were not consistently supported by significant pairwise differences. For instance, LRT showed significant effects compared with the control group [SMD = 1.43, 95% CI: 0.14–2.73] and AEWR [SMD = 1.98, 95% CI: 0.30–3.67], whereas its superiority over other interventions such as Resistance Training (RT) [SMD = 0.26, 95% CI: −0.88 to 1.41] was not statistically significant. Similarly, although LBFRT ranked highly, its comparisons with RT [SMD = 0.27, 95% CI: −1.32 to 1.86] and Upper Limb Resistance Training (URT) [SMD = −0.35, 95% CI: −1.80 to 1.09] had wide or overlapping CIs (Supplementary Table S8.1, Figure 4). Overall, the certainty of evidence for most comparisons was low (Supplementary Table S4.1).

#### 3.4.2. Obese Population

A total of five randomized controlled trials examined the effects of exercise interventions on VEGF levels in obese individuals (Supplementary Figure S7.1). The inconsistency test indicated no evidence of global inconsistency (*p* = 0.592) (Supplementary S5), and a consistency model was therefore applied. Since no closed loops were formed, node-splitting analysis was not required. SUCRA rankings (Supplementary Figure S10.1) suggested that Aerobic and Resistance Training under Hypoxia (AWRH, SUCRA = 74.7) and High-intensity Aerobic Exercise (HAE, SUCRA = 55.3) were most likely to be effective, while resistance training (RT, SUCRA = 31.5) ranked lowest. However, none of the pairwise comparisons between interventions reached statistical significance, and most 95% confidence intervals were wide and overlapped substantially (Supplementary Table S8.2, Supplementary Figure S9.1). For example, AWRH showed only a non-significant trend compared with CG [SMD = 0.86, 95% CI: −2.65 to 4.37], and HAE versus CG [SMD = 0.20, 95% CI: −0.91 to 1.31] also failed to demonstrate a significant effect. Overall, the certainty of evidence for the obese population was low (Supplementary Table S4.2).

#### 3.4.3. Patient Populations

A total of 12 randomized controlled trials evaluated the effects of exercise interventions on VEGF levels in patient populations (Supplementary Figure S7.2). The inconsistency test indicated no global inconsistency (*p* = 0.954), and a consistency model was applied. Since closed loops were present, node-splitting analysis was conducted and showed no significant local inconsistency (all *p* > 0.05) (Supplementary S5 and Table S6). SUCRA rankings (Supplementary Figure S10.2) suggested that Upper Limb Resistance Training (URT, SUCRA = 80.6) and TaiJi With Resistance (TWR, SUCRA = 74.9) were most likely to be effective, whereas the control group (CG, SUCRA = 27.3) ranked lowest. However, none of the pairwise comparisons between interventions achieved statistical significance, and most 95% confidence intervals were wide and overlapped considerably (Supplementary Table S8.3, Supplementary Figure S9.2). For instance, URT showed only a non-significant trend compared with CG [SMD = 1.54, 95% CI: −1.46 to 4.55], and TWR compared with CG [SMD = 1.25, 95% CI: −1.99 to 4.49] also did not reach significance. Overall, the certainty of evidence for the patient population was low (Supplementary Table S4.3).

### 3.5. Dose–Response Meta-Analysis

**Figure 5:**
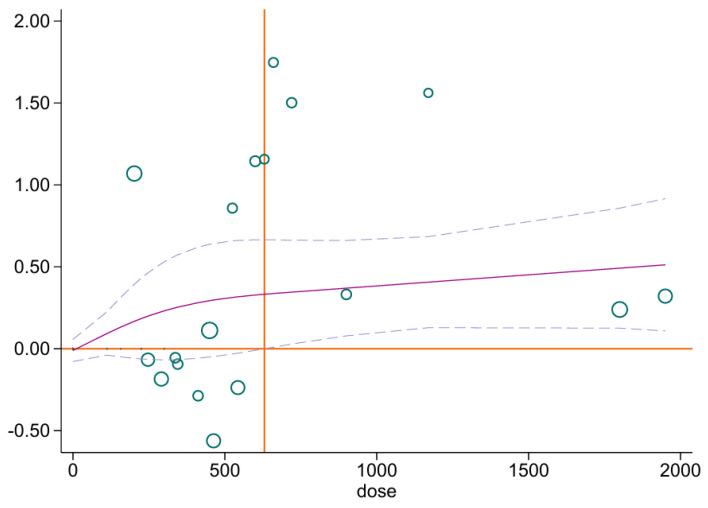
Dose-response results of healthy population. The solid and dashed purple lines illustrate the fitted dose–response curve and its 95% confidence interval, respectively. The orange horizontal line indicates the null effect (effect size = 0), whereas the vertical orange line marks the threshold dose for exercise.”

Dose–response analyses revealed distinct patterns across populations: in healthy individuals, VEGF levels increased linearly with exercise volume, with significant effects above ~630 METs-min/week and a peak at ~1950 METs-min/week (Figure 5); in obese individuals, no significant association was observed (Supplementary Figure S14.1); and in clinical populations, a U-shaped curve was detected, with significant improvements between 756 and 990 METs-min/week and a peak around 756 METs-min/week (Supplementary Figure S14.2).

In Figure 5, the solid and dashed purple lines illustrate the fitted dose–response curve and its 95% confidence interval, respectively. The orange horizontal line indicates the null effect (effect size = 0), whereas the vertical orange line marks the threshold dose for exercise.

### 3.6. Meta-Regression

Exploratory meta-regression analyses were conducted to examine potential moderators of VEGF responses (Supplementary Table S12). In the healthy population, age was identified as a significant factor (β = −0.01, SE = 0.0068, *p* = 0.012), indicating that younger cohorts tended to show greater VEGF increases. In the obese population, none of the tested covariates, including age, sex distribution, assay method, or biological sample source, reached statistical significance, although age showed a borderline association (*p* = 0.084). In patients with chronic conditions, the detection method emerged as a significant moderator (β = 0.48, SE = 0.23, *p* = 0.036), suggesting that VEGF effect sizes varied depending on whether ELISA, multiplex, or electrochemiluminescence assays were applied. No other covariates showed significant associations across populations. These findings are further illustrated in the network meta-regression bubble plots (Supplementary S13), which display the distribution of effect sizes by covariate levels and study precision.

### 3.7. Publication Bias

**Figure 6:**
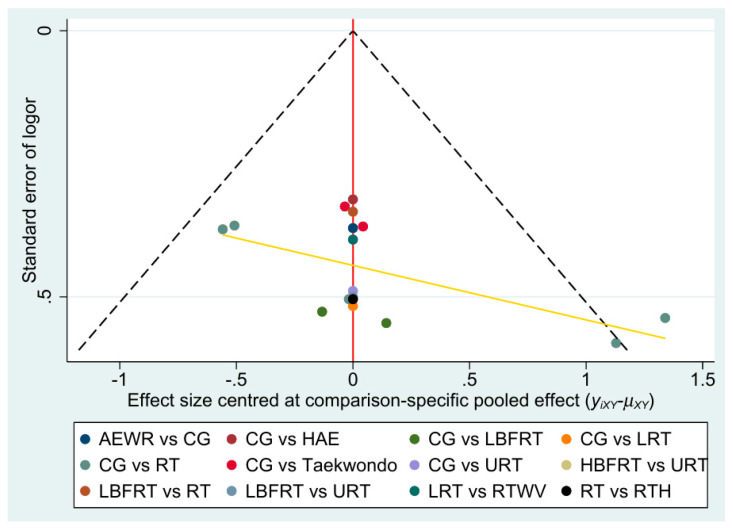
Funnel plots of healthy population.

Funnel plots for the healthy population (Figure 6) appeared largely symmetrical, with no clear evidence of small-study effects, although minor deviations were noted in a few comparisons. In the obese population (Supplementary Figure S11.1), the distribution was also balanced without marked asymmetry. For the patient population (Supplementary Figure S11.2), the plot suggested an approximately symmetrical pattern, despite some scattered points at the margins. Overall, these findings indicate no strong evidence of publication bias across populations.

## 4. Discussion

### 4.1. Main Findings

This study employed a network meta-analysis to comprehensively evaluate the effects of different exercise modalities and doses on VEGF levels across diverse populations. The main findings were as follows: (1) among healthy individuals, LRT was the most effective intervention for enhancing VEGF levels, with more pronounced improvements observed when the weekly exercise dose reached approximately 600 METs-min/week or above; (2) in individuals with obesity, aerobic exercise combined with resistance training under hypoxic conditions demonstrated relatively greater benefits, although no statistically significant differences were detected across exercise types or doses; and (3) in patients with cardiovascular or neurodegenerative diseases, Upper Limb Resistance Training (URT) appeared to exert the strongest effect on VEGF, with the greatest improvements occurring when weekly exercise doses were maintained at approximately 756–950 METs-min/week, despite the absence of significant differences among intervention types. Collectively, these findings provide important evidence to inform the development of more precise and individualized exercise prescriptions in clinical and rehabilitation settings.

### 4.2. VEGF Responses to Resistance Training in Healthy Individuals

Relative hypoxia during high-load contractions activates hypoxia-inducible factor-1α (HIF-1α), which in turn enhances VEGF expression and promotes capillary angiogenesis in skeletal muscle; this mechanism has been consistently reported in exercise studies [34]. In addition, mechanical tension and shear stress generated during repeated contractions are hypothesized to contribute to eNOS/NO signaling, thereby facilitating vascular reactivity and upregulating VEGF expression [35,36]. From a metabolic perspective, lactate accumulation and the resulting acidic milieu may activate MAPK/ERK pathways to further enhance VEGF expression, a mechanism that has been verified in models of choroidal neovascularization [37]. Although Gpr132 has been identified as a pH-sensitive receptor capable of sensing lactate/acidic signals and regulating macrophage responses in tumor models, its relevance for exercise-induced angiogenesis remains speculative [38]. Moreover, recent findings indicate that low-intensity resistance training combined with blood flow restriction can enhance eNOS and VEGF expression and improve endothelial function, which supports the possibility of interactions between shear stress and metabolic stimuli in vascular adaptations [35].

### 4.3. VEGF Modulation by Hypoxic Combined Training in Obesity

Our results suggest that aerobic and resistance training under hypoxic conditions may be the most effective strategy to enhance VEGF levels in obese individuals, although no significant differences were observed across METs-min/week. While direct evidence is limited, this finding is consistent with previous reports showing that hypoxia markedly upregulates VEGF in various tissues [39] and that combined aerobic–resistance training improves endothelial function partly through VEGF-mediated pathways [40]. Obesity is characterized by chronic low-grade inflammation driven by excessive adipose tissue, which secretes pro-inflammatory cytokines such as TNF-α and IL-6, thereby impairing insulin signaling and inducing insulin resistance [41,42]. Under hypoxic conditions, stabilization of HIF-1α promotes its binding to hypoxia response elements within the VEGF promoter, which could enhance VEGF transcription and prolong VEGF mRNA half-life through interactions with RNA-binding proteins such as HuR [39,43,44]. In parallel, combined aerobic and resistance training is known to reduce fat mass, improve insulin sensitivity, and modulate adipokines, changes that may secondarily normalize VEGF secretion and vascular remodeling [45,46,47,48]. Furthermore, obesity is associated with heightened sympathetic nervous system (SNS) activity, and regular exercise can attenuate SNS tone, potentially contributing to vascular regulation and angiogenic signaling [49,50,51].

### 4.4. VEGF Enhancement Through Upper-Limb Resistance Training in Patients with Chronic Diseases

This study demonstrated that upper-limb resistance training was most effective in increasing VEGF levels, with the optimal response observed at a weekly training dose of 756–950 METs-min/week. This finding aligns with emerging evidence that resistance training enhances skeletal muscle angiogenesis through upregulation of VEGF and its receptors [52]. In cardiovascular rehabilitation, upper-limb resistance exercise might deliver sufficient mechanical and hemodynamic stimuli while posing relatively lower cardiometabolic demand, thus supporting angiogenic signaling in patients with limited exercise tolerance [53,54]. Although upper-limb muscles are smaller in mass, the mechanical and hemodynamic stimuli during resistance contractions are adequate to induce endothelial remodeling and capillarization [55]. In addition, resistance training acutely elevates anabolic hormones such as IGF-1 and testosterone, which could play a role in vascular adaptation and VEGF signaling pathways [54,56]. These adaptations have been associated with improvements in vasodilatory function, microcirculation, and cardiovascular risk factors in patients with endothelial dysfunction [52]. For psychiatric populations, upper-limb resistance training may also provide a practical and safe modality, offering mechanobiological stimuli without excessive cardiopulmonary strain. Moreover, resistance training has been shown to attenuate sympathetic nervous system activity and systemic inflammation while modulating neuroendocrine markers (e.g., cortisol, norepinephrine, serotonin) [54,56]. which may indirectly influence VEGF expression and vascular health.

### 4.5. Clinical Implications

The findings of this study hold important clinical value for designing tailored exercise rehabilitation programs across different populations. For healthy individuals, moderate-to-high intensity lower-limb resistance training is recommended to optimize angiogenesis. In obese patients, combining hypoxic exposure with aerobic and resistance training may improve both metabolic and vascular health. For individuals with cardiovascular or mental disorders, moderate-intensity, low-risk upper-limb resistance training should be prioritized. Proper regulation of training dose (e.g., 600 or 750–950 METs-min/week) not only facilitates VEGF upregulation but also helps avoid excessive cardiovascular stress. These strategies can inform personalized exercise prescriptions aimed at promoting vascular remodeling, enhancing endothelial function, and improving outcomes in chronic disease rehabilitation.

## 5. Limitations and Future Directions

This study offers several strengths. It is among the first to systematically evaluate the effects of multiple exercise modalities on VEGF levels through both network and dose–response meta-analyses, providing a comprehensive synthesis of current evidence. The inclusion of both healthy individuals and patients with chronic non-cancer conditions enhances the generalizability of the findings, and standardized data extraction together with rigorous risk of bias assessment ensures methodological robustness. Nevertheless, several limitations should be acknowledged. First, the sources of VEGF measurements (serum vs. plasma) and assay methods varied across studies, which may have introduced heterogeneity. Second, given the limited number of trials, dose–response analyses were conducted using a pooled framework rather than modality-specific modeling, potentially obscuring differential patterns across exercise types. Third, key demographic variables such as age, sex, and race were inconsistently reported, limiting the ability to perform detailed subgroup analyses. Fourth, exercise doses were not always directly reported but estimated from frequency, duration, and intensity data, which may have introduced measurement error into the dose–response models. Fifth, although cancer populations were included in the initial protocol, insufficient eligible trials prevented their inclusion in the analysis. Finally, excessive upregulation of VEGF may also cause adverse effects, particularly under intensive exercise training or when combined with VEGF-modulating pharmacological agents, which should be considered in future investigations. Future research should prioritize larger and more diverse cohorts, longer follow-up durations, consistent reporting of demographic and intervention details, and the integration of objective monitoring technologies (e.g., wearables) to enhance the precision of exercise dose quantification and clarify the role of exercise in modulating VEGF.

## 6. Conclusions

By means of a network meta-analysis, this study found that high-intensity lower-limb resistance training (≥600 METs-min/week) may be associated with greater improvements in VEGF levels among healthy populations. In obese populations, aerobic plus resistance training under hypoxic conditions appeared to be more beneficial, although differences in exercise volume did not show statistically significant effects. For individuals with cardiovascular/cerebrovascular diseases or mental disorders, upper-limb resistance training within 756–950 METs-min/week showed a potentially greater effect on elevating VEGF levels. These findings highlight the potential importance of tailoring exercise type and intensity to different populations to better support angiogenic adaptations. To further improve individualized intervention outcomes, large-scale, long-term prospective studies are warranted, ideally incorporating wearable devices and mobile health applications for real-time monitoring and feedback. Such approaches may help optimize dose–response analyses and clarify appropriate exercise prescriptions for different subgroups. For individuals with other chronic conditions or specific physiological characteristics, regular monitoring and individualized adjustments may also play a critical role in ensuring safety and efficacy. Overall, the current evidence suggests that precise exercise prescriptions may hold promise for clinical and rehabilitation applications.

## Figures and Tables

**Table 1:** Summary of Exercise Interventions, VEGF Measurement, and Key Findings Across Populations.

Population Group	No. of Studies	Total Participants	Age Range	Interventions	Dose Range	VEGF Assays	Key Findings
Healthy adults	11	313	18–80	RT, LBFRT, AEWR, Taekwondo, RTWV	200–1950	Plasma/Serum; ELISA	Lower-limb RT and Taekwondo significantly increased VEGF; effect most evident ≥600 METs-min/week
Obese individuals	5	321	20–70	CAREX, AEWR, HAE, RIPC	720–3300	Plasma/Serum; ELISA (RayBiotech, DRG, etc.)	Combined aerobic + resistance (esp. hypoxic) produced the largest VEGF gains; no clear dose–response
Clinical populations (CVD, HF, HTN, MetS, T2D, AD, Parkinson’s, Depression, Cognitive impairment)	12	482	40–80	MAE, HAE, HIIT, URT, Taiji, AETOT	450–1600	Serum/Plasma; ELISA, ECL	Moderate RT and AE improved VEGF; optimal dose ~756–950 METs-min/week in clinical subgroups
Total	28	1138	18–80	8 intervention types	200–3300	Mostly ELISA-based	Exercise effects are population- and dose-specific

## Data Availability

Date are contained within the article and supplementary materials.

## Supplementary Materials

The following supporting information can be downloaded at: https://www.mdpi.com/article/10.3390/biomedicines13102548/s1, Supplemental Table S1 Search Strategies; Supplemental Table S2 Basic information included in the study; Supplemental Table S3 Risk of bias summary; Supplementary S4-GRADE Assessment for Network Evidence; Supplementary S5 Global Inconsistency Test; Supplemental Table S6 Node splitting method-local inconsistency; Supplementary S7 Network Plots for All Outcomes; Supplemental S8 League table; Supplemental S9 Pairwise comparison forest plot; Supplemental S10 Overall SUCRA Rankings; Supplementary S11 Funnel Plots for Publication Bias; Supplementary S12 Meta-Regression Results; Supplementary S13 Network Meta-Regression Bubble Plots; Supplementary S14 Dose–response results. References [57,58,59,60,61,62,63,64,65,66,67,68,69,70,71,72,73,74,75,76,77,78,79,80,81,82,83] are cited in the supplementary materials.

## Abbreviations

The following abbreviations are used in this manuscript:

LBFRT	Low-intensity Blood Flow Restriction Resistance Training
HBFRT	High-intensity Blood Flow Restriction Resistance Training
RT	Resistance Training
AEWR	Aerobic Exercise with Resistance
AWRH	Aerobic and Resistance Training under Hypoxia
MAE	Moderate-intensity Aerobic Exercise
HAE	High-intensity Aerobic Exercise
URT	Upper Limb Resistance Training
LRT	Lower Limb Resistance Training
RTWV	Resistance Training with Vibration
AEH	Aerobic Exercise under Hypoxia
TWR	TaiJi with Resistance
RIPC	Remote Ischemic Preconditioning Aerobic Exercise
RTH	Resistance Training under Hypoxia
AETOT	Aerobic Exercise with Task-Oriented Training
