# Supplementary material for: The Optimal Type and Dose of Exercise Interventions on VEGF Levels in Healthy Individuals, as Well as Obesity and Chronic Disease Populations: A Network Meta-Analysis
Source: Biomedicines. 2025 Oct 19;13(10):2548. doi: 10.3390/biomedicines13102548 (PMC12562027; doi:10.3390/biomedicines13102548)
Supplement: Supplementary file 1 [file biomedicines-13-02548-s001.zip › biomedicines-3884182-supplementary.pdf]

Supplemental

Supplemental Table S1 Search Strategies....................2
Supplemental Table S2 Basic information included in the study....................6
Supplemental Table S3 Risk of bias summary....................11
Supplementary S4 GRADE Assessment for Network Evidence....................13
Supplementary S5 Global Inconsistency Test....................19
Supplemental Table S6 Node splitting method-------local inconsistency....................20
Supplementary S7 Network Plots for All Outcomes....................22
Supplemental S8 League table....................23
Supplemental S9 Pairwise comparison forest plot....................25
Supplemental S10 Overall SUCRA Rankings....................26
Supplementary S11 Funnel Plots for Publication Bias....................27
Supplementary S12 Meta-Regression Results....................28
Supplementary S13 Network Meta-Regression Bubble Plots....................29
Supplementary S14 Dose-response results....................32

Supplemental Table S1 Search Strategies

**Database: PubMed (869)**	
#1	"Exercise"[Mesh]
#2	((Exercises[Title/Abstract]) OR (Exercise, Physical[Title/Abstract])) OR (Exercises, Physical[Title/Abstract])) OR (Physical Exercise[Title/Abstract])) OR (Physical Exercises[Title/Abstract])) OR (Exercise, Aerobic[Title/Abstract])) OR (Aerobic Exercise[Title/Abstract])) OR (Aerobic Exercises[Title/Abstract])) OR (Exercises, Aerobic[Title/Abstract])) OR (Exercise, Isometric[Title/Abstract])) OR (Exercises, Isometric[Title/Abstract])) OR (Isometric Exercises[Title/Abstract])) OR (Isometric Exercise[Title/Abstract])) OR (Acute Exercise[Title/Abstract])) OR (Acute Exercises[Title/Abstract])) OR (Exercise, Acute[Title/Abstract])) OR (Exercises, Acute[Title/Abstract])) OR (Exercise Training[Title/Abstract])) OR (Exercise Trainings[Title/Abstract])) OR (Training, Exercise[Title/Abstract])) OR (Trainings, Exercise[Title/Abstract])) OR (Physical Activity[Title/Abstract])) OR (Activities, Physical[Title/Abstract])) OR (Activity, Physical[Title/Abstract])) OR (Physical Activities[Title/Abstract])) OR (Resistance Training[Title/Abstract])) OR (Training, Resistance[Title/Abstract])) OR (Strength Training[Title/Abstract])) OR (Training, Strength[Title/Abstract])) OR (Strengthening Programs, Weight-Lifting[Title/Abstract])) OR (Strengthening Program, Weight-Lifting[Title/Abstract])) OR (Exercise Programs, Weight-Lifting[Title/Abstract])) OR (Exercise Program, Weight-Lifting[Title/Abstract])) OR (Weight-Bearing Strengthening Program[Title/Abstract])) OR (Strengthening Programs, Weight-Bearing[Title/Abstract])) OR (Strengthening Program, Weight-Bearing[Title/Abstract])) OR (Weight Bearing Strengthening Program[Title/Abstract])) OR (Weight-Bearing Strengthening Programs[Title/Abstract])) OR (Weight-Bearing Exercise Program[Title/Abstract])) OR (Exercise Programs, Weight-Bearing[Title/Abstract])) OR (Exercise Program, Weight-Bearing[Title/Abstract])) OR (Weight Bearing Exercise Program[Title/Abstract])) OR (Weight-Bearing Exercise Programs[Title/Abstract])
#3	"Vascular Endothelial Growth Factors"[Mesh]
#4	(VEGFs[Title/Abstract]) OR (VEGF[Title/Abstract])
#5	("Exercise"[Mesh]) OR (((Exercises[Title/Abstract]) OR (Exercise, Physical[Title/Abstract])) OR (Exercises, Physical[Title/Abstract])) OR (Physical Exercise[Title/Abstract])) OR (Physical Exercises[Title/Abstract])) OR (Exercise, Aerobic[Title/Abstract])) OR (Aerobic Exercise[Title/Abstract])) OR (Aerobic Exercises[Title/Abstract])) OR (Exercises, Aerobic[Title/Abstract])) OR (Exercise, Isometric[Title/Abstract])) OR (Exercises, Isometric[Title/Abstract])) OR (Isometric Exercises[Title/Abstract])) OR (Isometric Exercise[Title/Abstract])) OR (Acute Exercise[Title/Abstract])) OR (Acute Exercises[Title/Abstract])) OR (Exercise, Acute[Title/Abstract])) OR (Exercises, Acute[Title/Abstract])) OR (Exercise

	Training[Title/Abstract])) OR (Exercise Trainings[Title/Abstract])) OR (Training, Exercise[Title/Abstract])) OR (Trainings, Exercise[Title/Abstract])) OR (Physical Activity[Title/Abstract])) OR (Activities, Physical[Title/Abstract])) OR (Activity, Physical[Title/Abstract])) OR (Physical Activities[Title/Abstract])) OR (Resistance Training[Title/Abstract])) OR (Training, Resistance[Title/Abstract])) OR (Strength Training[Title/Abstract])) OR (Training, Strength[Title/Abstract])) OR (Strengthening Programs, Weight-Lifting[Title/Abstract])) OR (Strengthening Program, Weight-Lifting[Title/Abstract])) OR (Exercise Programs, Weight-Lifting[Title/Abstract])) OR (Exercise Program, Weight-Lifting[Title/Abstract])) OR (Weight-Bearing Strengthening Program[Title/Abstract])) OR (Strengthening Programs, Weight-Bearing[Title/Abstract])) OR (Strengthening Program, Weight-Bearing[Title/Abstract])) OR (Weight Bearing Strengthening Program[Title/Abstract])) OR (Weight-Bearing Strengthening Programs[Title/Abstract])) OR (Weight-Bearing Exercise Program[Title/Abstract])) OR (Exercise Programs, Weight-Bearing[Title/Abstract])) OR (Exercise Program, Weight-Bearing[Title/Abstract])) OR (Weight Bearing Exercise Program[Title/Abstract])) OR (Weight-Bearing Exercise Programs[Title/Abstract]))
#6	("Vascular Endothelial Growth Factors"[Mesh]) OR ((VEGFs[Title/Abstract]) OR (VEGF[Title/Abstract]))
#7	(("Exercise"[Mesh]) OR (((Exercises[Title/Abstract]) OR (Exercise, Physical[Title/Abstract])) OR (Exercises, Physical[Title/Abstract])) OR (Physical Exercise[Title/Abstract])) OR (Physical Exercises[Title/Abstract])) OR (Exercise, Aerobic[Title/Abstract])) OR (Aerobic Exercise[Title/Abstract])) OR (Aerobic Exercises[Title/Abstract])) OR (Exercises, Aerobic[Title/Abstract])) OR (Exercise, Isometric[Title/Abstract])) OR (Exercises, Isometric[Title/Abstract])) OR (Isometric Exercises[Title/Abstract])) OR (Isometric Exercise[Title/Abstract])) OR (Acute Exercise[Title/Abstract])) OR (Acute Exercises[Title/Abstract])) OR (Exercise, Acute[Title/Abstract])) OR (Exercises, Acute[Title/Abstract])) OR (Exercise Training[Title/Abstract])) OR (Exercise Trainings[Title/Abstract])) OR (Training, Exercise[Title/Abstract])) OR (Trainings, Exercise[Title/Abstract])) OR (Physical Activity[Title/Abstract])) OR (Activities, Physical[Title/Abstract])) OR (Activity, Physical[Title/Abstract])) OR (Physical Activities[Title/Abstract])) OR (Resistance Training[Title/Abstract])) OR (Training, Resistance[Title/Abstract])) OR (Strength Training[Title/Abstract])) OR (Training, Strength[Title/Abstract])) OR (Strengthening Programs, Weight-Lifting[Title/Abstract])) OR (Strengthening Program, Weight-Lifting[Title/Abstract])) OR (Exercise Programs, Weight-Lifting[Title/Abstract])) OR (Exercise Program, Weight-Lifting[Title/Abstract])) OR (Weight-Bearing Strengthening Program[Title/Abstract])) OR (Strengthening Programs, Weight-Bearing[Title/Abstract])) OR (Strengthening Program, Weight-Bearing[Title/Abstract])) OR (Weight Bearing Strengthening Program[Title/Abstract])) OR (Weight-Bearing Strengthening Programs[Title/Abstract])) OR (Weight-Bearing Exercise Program[Title/Abstract])) OR (Exercise Programs, Weight-Bearing[Title/Abstract])) OR (Exercise Program, Weight-Bearing[Title/Abstract])) OR (Weight Bearing Exercise

	Program[Title/Abstract])) OR (Weight-Bearing Exercise Programs[Title/Abstract]))) AND (("Vascular Endothelial Growth Factors"[Mesh]) OR ((VEGFs[Title/Abstract]) OR (VEGF[Title/Abstract])))
**Database: Web of science (5463)**	
	Ts=（Exercise or Exercises or Exercise, Physical or Exercises, Physical or Physical Exercise or Physical Exercises or Exercise, Aerobic or Aerobic Exercise orAerobic Exercises or Exercises, Aerobic or Exercise, Isometric or Exercises, Isometric or Isometric Exercises or Isometric Exercise or Acute Exercise or Acute Exercises or Exercise, Acute or Exercises, Acute or Exercise Training or Exercise Trainings or Training, Exercise or Trainings, Exercise or Physical Activity or Activities, Physical or Activity, Physical or Physical Activities or Resistance Training or Training, Resistance or Strength Training or Training, Strength or Strengthening Programs, Weight-Lifting or Strengthening Program, Weight-Lifting or Exercise Programs, Weight-Lifting or Exercise Program, Weight-Lifting or Weight-Bearing Strengthening Program or Strengthening Programs, Weight-Bearing or Strengthening Program, Weight-Bearing or Weight Bearing Strengthening Program or Weight-Bearing Strengthening Programs or Weight-Bearing Exercise Program or Exercise Programs, Weight-Bearing or Exercise Program, Weight-Bearing or Weight Bearing Exercise Program or Weight-Bearing Exercise Programs）and ts=（Vascular Endothelial Growth Factors OR VEGFs OR VEGF）
https://webofscience.clarivate.cn/wos/alldb/summary/e4763057-c2f4-43e6-bf71-3e363d1d0398-0144340567/relevance/1	
**Database: EMbase（2631）**	
#7	#5 AND #6
#6	#3 OR #4
#5	#1 OR #2
#4	'vascular endothelial growth factors':ti,ab OR 'vegfs':ti,ab OR 'vegf':ti,ab
#3	'vasculotropin'/exp
#2	'exercise':ti,ab OR 'physical exercise':ti,ab OR 'physical exercises':ti,ab OR 'exercise, physical':ti,ab OR 'exercises, physical':ti,ab OR 'exercise, aerobic':ti,ab OR 'aerobic exercise':ti,ab OR 'aerobic exercises':ti,ab OR 'exercises, aerobic':ti,ab OR 'exercise, isometric':ti,ab OR 'exercises, isometric':ti,ab OR 'isometric exercises':ti,ab OR 'isometric exercise':ti,ab OR 'acute exercise':ti,ab OR 'acute exercises':ti,ab OR 'exercise, acute':ti,ab OR 'exercises, acute':ti,ab OR 'exercise training':ti,ab OR 'exercise trainings':ti,ab OR 'training, exercise':ti,ab OR 'trainings, exercise':ti,ab OR 'physical activity':ti,ab OR 'activities, physical':ti,ab OR 'activity, physical':ti,ab OR 'physical activities':ti,ab OR 'resistance training':ti,ab OR 'training, resistance':ti,ab OR 'strength training':ti,ab OR 'training, strength':ti,ab OR 'strengthening programs, weight-lifting':ti,ab OR 'strengthening program, weight-lifting':ti,ab OR 'exercise programs, weight-lifting':ti,ab OR 'exercise program, weight-lifting':ti,ab OR 'weight-bearing strengthening program':ti,ab OR

	'strengthening programs, weight-bearing':ti,ab OR 'strengthening program, weight-bearing':ti,ab OR 'weight bearing strengthening program':ti,ab OR 'weight-bearing strengthening programs':ti,ab OR 'weight-bearing exercise program':ti,ab OR 'exercise programs, weight-bearing':ti,ab OR 'exercise program, weight-bearing':ti,ab OR 'weight bearing exercise program':ti,ab OR 'weight-bearing exercise programs':ti,ab
#1	'exercise'/exp
**Database:The Cochrane Library（397）**	
#1	MeSH descriptor: [exercise] explode all trees
#2	'exercise':ti,ab OR 'physical exercise':ti,ab OR 'physical exercises':ti,ab OR 'exercise, physical':ti,ab OR 'exercises, physical':ti,ab OR 'exercise, aerobic':ti,ab OR 'aerobic exercise':ti,ab OR 'aerobic exercises':ti,ab OR 'exercises, aerobic':ti,ab OR 'exercise, isometric':ti,ab OR 'exercises, isometric':ti,ab OR 'isometric exercises':ti,ab OR 'isometric exercise':ti,ab OR 'acute exercise':ti,ab OR 'acute exercises':ti,ab OR 'exercise, acute':ti,ab OR 'exercises, acute':ti,ab OR 'exercise training':ti,ab OR 'exercise trainings':ti,ab OR 'training, exercise':ti,ab OR 'trainings, exercise':ti,ab OR 'physical activity':ti,ab OR 'activities, physical':ti,ab OR 'activity, physical':ti,ab OR 'physical activities':ti,ab OR 'resistance training':ti,ab OR 'training, resistance':ti,ab OR 'strength training':ti,ab OR 'training, strength':ti,ab OR 'strengthening programs, weight-lifting':ti,ab OR 'strengthening program, weight-lifting':ti,ab OR 'exercise programs, weight-lifting':ti,ab OR 'exercise program, weight-lifting':ti,ab OR 'weight-bearing strengthening program':ti,ab OR 'strengthening programs, weight-bearing':ti,ab OR 'strengthening program, weight-bearing':ti,ab OR 'weight bearing strengthening program':ti,ab OR 'weight-bearing strengthening programs':ti,ab OR 'weight-bearing exercise program':ti,ab OR 'exercise programs, weight-bearing':ti,ab OR 'exercise program, weight-bearing':ti,ab OR 'weight bearing exercise program':ti,ab OR 'weight-bearing exercise programs':ti,ab
#3	MeSH descriptor: [vasculotropin] explode all trees
#4	'vascular endothelial growth factors':ti,ab OR 'vegfs':ti,ab OR 'vegf':ti,ab
#5	#1 OR #2
#6	#3 OR #4
#7	#5 OR #6

Supplemental Table S2 Basic information included in the study

First author	Published	Nation	Number of people	Gender composition	Age	Population types	VEGF source	Assay method	Sampling timing	Intervention session duration	Intervention frequency	Intervention intensity	Intervention period	Intervention measures	Units of VEGF	Weekly MET-minutes
1.Saman Sharif[1]	2020	Iran	8<br>8<br>8<br>8<br>8	Males only (100%)	21.25±1.9<br>18.75±1.63<br>18.75±1.46<br>20.12±2.19<br>22.69±0.69	Healthy population	Plasma	ELISA (Bender MedSystems, Vienna, Austria)	Morning (08:00–10:00), fasting blood samples	50min<br>30min<br>50min<br>30min<br>0	3 times a week<br>3 times a week (Twice a day)<br>3 times a week<br>3 times a week (Twice a day)<br>0	20-30% 1RM<br>20-30% 1RM<br>70-80% 1RM<br>70-80% 1RM<br>0	6 weeks	LBFRT<br>LBFRT<br>RT<br>RT<br>CG	pg/ml	525<br>630<br>900<br>1170<br>0
13.Francesco P. Boeno[2]	2020	USA	15<br>15<br>12	19 Males, 23 Females (45.2% male)	30-59	Hypertensive patients	Plasma	ELISA (BosterBio, Pleasanton, CA, USA)	Morning (07:00–09:00), after 10–12 h overnight fast	60min<br>45min<br>0	3 times a week	60-80 HR<br>50-80% 1RM<br>0	12 weeks	MAE<br>RT<br>CG	pg/ml	1260<br>756<br>0
16.Wonil Park[3]	2024	South Korea	12<br>12	Females only (0%)	68.08±0.9<br>67.83±1.03	Obese population	Serum	ELISA (Quantikine, R&D Systems, Minneapolis, MN, USA)	Morning (~08:00), after 12 h fasting	155 min<br>155 min	3 times a week<br>3 times a week	60-70%HR and OMNI:7-8<br>60-70%HR and OMNI:7-8	12 weeks	AEWR<br>AWRH	pg/mL	2160<br>2484
17.Michael Despeghel[4]	2021	Switzerland	30<br>10	21 Males, 19 Females (52.5% male)	70.4±5.3<br>69.8±4.4	Healthy population	Plasma	Multiplex ELISA (Bio-Techne, UK; Luminex Magpix, USA)	Morning (08:00–09:00), fasting, at rest	50min<br>0	2 times a week<br>0	60% 1RM<br>0	6 weeks	AEWR<br>CG	pg/mL	463<br>0
19.Marzena	2019	Poland	22<br>17	Females only (0%)	28-62	Obese population	Plasma	ELISA (DRG Internatio	Morning, after 12 h overnight	60min	3 times a week	60-80% HR<br>60-80% HR<br>50-60 1RM	3 months	MAE<br>AEWR	pg/mL	945<br>1110

Ratajczak[5]								nal, USA)	fast, resting state	60min	3 times a week					
22.DM Croymans[6]	2014	USA	28<br>8	Males only (100%)	21.5(20.0–23.0)<br>22.0(20.8–22.8)	Obese population	Serum	ELISA (R&D Systems, USA)	Outpatient visits (07:30), fasting after overnight fast (~3.5 h visit)	60min	3 times a week<br>0	40-100%1RM<br>0	12 weeks	RT<br>CG	pg/mL	1260<br>0
23.Michihiro Kon[7]	2014	Japan	7<br>9	Males only (100%)	28.4±4.8<br>28.2±3.7	Healthy population	Plasma	ELISA (R&D Systems, USA)	Morning (08:30–11:00), after overnight fast, 30 min rest	15min<br>15min	2 times a week<br>2 times a week	70% 1RM<br>70% 1RM	8 weeks	RT<br>RTH	pg/ml	300<br>345
24.P. Farzanegi[8]	2020	Iran	10<br>10<br>10<br>10	Males only (100%)	24.15±2.77<br>21±0.79<br>21.2±0.76<br>22.26±2.23	Healthy population	Serum	ELISA (Cusabio, Wuhan, China; Cat No. CSB-E11718h)	Morning, after 10–12 h overnight fast; baseline and 8 weeks post-intervention	40min<br>40min<br>40min<br>0	3 times a week<br>3 times a week<br>3 times a week<br>0	70% 1RM<br>70% 1RM<br>70% 1RM<br>0	8 weeks	URT<br>LRT<br>RT<br>CG	ng/ml	600<br>720<br>660<br>0
25.Yan Zhao[9]	2020	China	8<br>8<br>8	Males only (100%)	19±1<br>20±1<br>19±1	Healthy population	Plasma	ELISA (BMS277, Invitrogen, San Diego, CA, USA)	Fasting morning; at rest and immediately post-exercise	15min<br>15min<br>15min	5 times a week<br>5 times a week<br>5 times a week	30% 1RM<br>30% 1RM<br>30% 1RM	8 weeks	URT<br>LBFRT<br>HBFRT	pg/ml	225<br>337<br>412
30.Smita DuttaRoy[10]	2015	Sweden	24<br>21	37 Males, 8 Females (82.2% male)	62(58-68)<br>66(62-71)	CAD patients	Serum	High-sensitivity ELISA (Quantikine, R&D Systems)	Morning, after overnight fast and 15 min rest	30min<br>0	(3 resistance, 5 aerobic)/week<br>0	Borg RPE 13-15 and 75 1RM<br>0	8 weeks	AEWR<br>CG	%	1185<br>0
31.Ryosuke Shimizu[11]	2016	Japan	20<br>20	33 Males, 7 Females (82.5% male)	72±4<br>70±4	Healthy population	Plasma	Quantikine ELISA (R&D Systems)	Morning, fasting	15min<br>15min	3 times a week<br>3 times a week	20% 1RM<br>20% 1RM	4 weeks	LBFRT<br>RT	pg/ml	202<br>112
42Kieran J. Marston[12]	2019	Australia	14<br>15<br>15	8 Males, 36 Females (18.2% male)	55.2±6.8<br>58.0±5.5	Healthy population	Serum	DuoSet ELISA (R&D Systems)	Morning (06:00–09:00), 12 h	40-45min	2 times a week<br>2 times a week	85% 1RM<br>70% 1RM<br>0	12 weeks	RT<br>RT<br>CG	pg/ml	543<br>291<br>0

					59.1±7.4				overnight fast;	30min<br>0	0					
43.AsaBeijer[13]	2013	Germany	13<br>13	Males only (100%)	23.4±0.39<br>24.3±0.92	Healthy population	Serum	ELISA (R&D Systems)	At baseline and final training day; participants consumed standardized breakfast 2 h prior	9min<br>9min	2-3 times a week<br>2-3 times a week	80% 1RM<br>80% 1RM	6 weeks	LRT<br>RTWV	pg/ml	157<br>247
52.Anna Pedrinolla[14]	2020	Italy	20<br>19	13 Males, 26 Females (33.3% male)	79±7<br>79±9	AD patients	Plasma	ELISA (DRG International Inc., NJ, USA)	Morning (09:00–10:00), after 12 h overnight fast; no alcohol/caffeine/exercise 12 h prior	90min<br>0	3 times a week<br>0	70% HR and 85 1RM<br>0	6 months	AEWR<br>CG	pg/ml	855<br>0
54.Chia-Liang Tsai[15]	2019	China	19<br>18<br>18	17 Males, 38 Females (30.9% male)	66±7.68<br>65.44±6.76<br>65.17±7	Cognitive impairment patients	Serum	Luminex 200 system (Millipore, USA)	Blood drawn (10 mL), processed and stored at −80 ° C	40min<br>40min<br>0	3 times a week<br>3 times a week<br>0	50-75% HR<br>50-75% 1RM<br>0	16 weeks	MAE<br>RT<br>CG	ng/ml	720<br>540<br>0
55.Su-Youn Cho[16]	2019	South Korea	19	Females only (0%)	68.89±4.16<br>69±4.41	Healthy population	Serum	Sandwich ELISA (R&D Systems, USA; #DVE00)	Not reported	60min	5 times a week<br>0	50-80%HR<br>0	16 weeks	Taekwondo<br>CG	pg/ml	1800<br>0
56.Su-Youn Cho[17]	2017	South Korea	15<br>15	18 Males, 12 Females (60.0% male)	11.2±0.77<br>11.33±0.72	Healthy population	Serum	Quantikine ELISA (R&D Systems, USA; #DVE00)	Not reported	60min<br>0	5 times a week<br>0	Borg RPE 11-15<br>0	16 weeks	Taekwondo<br>CG	pg/ml	1950<br>0
59.Sandra Erbs[18]	2010	Germany	18<br>19	Males only (100%)	60±11<br>62±10	Heart failure patients	Plasma	High-sensitivity ELISA (R&D Systems,	Morning, fasting; medication withdrawal 24 h prior	12.5 min	Initially 4.5 times/day for the first 3 weeks, then 1 time/day after discharge.	50-60% HR	12 weeks	MAE<br>CG	pg/ml	820<br>0

								Germany)			0					
61.Kim M. Huffman [19]	2008	USA	50<br>42<br>49<br>48	101 Males, 88 Females (53.4% male)	40-65	Obese population	Plasma	Bio-Plex Cytokine Assays (Bio-Rad, USA)	Not reported		0<br>12 min/week or 1200 kcal/week, VO_2 40-55%HR<br>12 min/week or 1200 kcal/week, VO_2 65-80%HR<br>20 min/week or 2000 kcal/week, VO_2 65-80%HR		6 months	CG<br>MAE<br>HAE<br>HAE	pg/ml	0<br>420<br>530<br>1200
63.Xueqiao Jiao[20]	2024	China	9	16 Males, 17 Females (48.5% male)	31.33±8.75<br>36.62±9.54<br>39.18±12.68	Obese population	Serum	ELISA (RayBiotech, USA)	Fasting ≥8 h, morning at rest; pre- and post-intervention	60min<br>60min<br>60min	5 times a week<br>5 times a week<br>5 times a week	＞70%HR<br>＞70%HR<br>＞70%HR	4 weeks	HAE<br>AEH<br>RIPC	pg/ml	1350<br>1740<br>3300
64.Jesper Krogh[21]	2014	Denmark	41<br>38	53 Males, 26 Females (67.1% male)	38.9±11.7<br>43.8±12.2	Depression patients	Serum	Quantikine ELISA (R&D Systems, USA)	Morning (08:00–10:00), overnight fast (water only), 5 min rest; pre- & post-intervention	45min<br>0	3 times a week<br>0	80%HR<br>0	3 months	HAE<br>CG	pg/ml	1080<br>0
65.Song LIN[22]	2014	China	10<br>10	17 Males, 3 Females (85.0% male)	66.5±5.1<br>67.2±13.8	CAD patients	Serum	ELISA (R&D Systems, USA)	Not reported	40min<br>0	5 times a week(Twice a day)<br>0	50-60 1RM<br>0	3 months	URT<br>CG	pg/ml	1600<br>0
66.Xiaoli Liu[23]	2024	China	16<br>16<br>16	29 Males, 19 Females (60.4% male)	60.6±5.7<br>61.4±3.4<br>61.3±4.9	T2D patients	Plasma	ELISA (East China Electronics; Nanjing Institute protocol)	Morning, after >8 h overnight fast; baseline & 24 weeks	60min<br>60min<br>60min	6 times a week(Taiji)<br>6 times a week (2 RT, 4 Taiji)<br>0	95-105 Times/minute<br>95-115 Times/minute<br>0	24 weeks	Taiji<br>TWR<br>CG	ng/ml	1260<br>1050<br>0
67.Anne Maass[24]	2016	Germany	21<br>19	18 Males, 22 Females (45.0% male)	68.4 ± 4.3	Healthy population	Serum	Quantikine ELISA (R&D Systems, USA; DVE00)	Baseline: morning fasting; post: morning or on last training day; stored at −80 ° C	30min	3 times a week<br>3 times a week	Borg RPE 10	3 months	HAE<br>CG	ng/ml	450<br>0
68.Knut Mai[25]	2020	Germany	12<br>9	Males only (100%)	57.0 (52.3–62.5)	MetS patients	Plasma	ELISA (R&D	Morning (08:00), after 12 h	60min	3 times a week	50-60% HR<br>50-60% HR	6 weeks	AEH<br>MAE	pg/ml	900<br>810

					58.0 (49.5–65.0)			Systems, UK)	overnight fast; pre- & post-intervention (euglycemic clamp)	60min	3 times a week					
69.Stefan Peter Mortensen[26]	2018	Denmark	10<br>11	13 Males, 8 Females (61.9% male)	57±9<br>53±8	T2D patients	Serum	Electrochemiluminescence assay (VEGF165 kit, MSD, USA)	Morning (08:00), after ≥10 h overnight fast; baseline & 11 weeks	50min<br>30min	3 times a week<br>3 times a week	50% HR<br>95% HR	11 weeks	MAE<br>HIIT	pg/ug	450<br>990
73.Fatih Soke[27]	2021	Turkey	15<br>14	19 Males, 10 Females (65.5% male)	57.07±8.18<br>58.07±8.91	Parkinson's disease patients	Serum	ELISA (Sunred Biological Technology, Shanghai, China)	Morning (08:00–09:00), fasting from 20:00 prior; pre- and post-intervention	30min+50min<br>30min	3 times a week<br>3 times a week	60-80% HR and Borg RPE 12-15<br>60-80% HR	8 weeks	AETOT<br>HAE	pg/ml	1335<br>585
75.Mona Mohamed Taha[28]	2016	Egypt	23<br>23	Females only (0%)	45-55	Hypertensive patients	Serum	Quantikine ELISA (R&D Systems, USA; DVE00)	Morning, after 12 h overnight fast; baseline & 24–36 h post-intervention (to avoid acute exercise effect)	40min<br>0	3 times a week<br>3 times a week	80-85% HR<br>0	10 weeks	HIIT<br>CG	pg/ml	1080<br>0

Supplemental Table S3 Risk of bias summary

Study	1	2	3	4	5	6	7	8	9	10	11	12	13
**Saman Sharif**	Y	U	Y	U	U	U	Y	Y	Y	Y	Y	Y	Y
**Francesco P. Boeno**	Y	U	Y	U	U	Y	Y	Y	Y	Y	Y	Y	Y
**Wonil Park**	Y	Y	Y	U	U	Y	Y	Y	Y	Y	Y	Y	Y
**Michael Despeghel**	Y	U	Y	U	U	Y	Y	Y	Y	Y	Y	Y	Y
**Marzena Ratajczak**	Y	U	Y	U	U	Y	Y	Y	Y	Y	Y	Y	Y
**DM Croymans**	Y	Y	Y	U	U	Y	Y	Y	Y	Y	Y	Y	Y
**Michihiro Kon**	Y	U	Y	U	U	Y	Y	Y	Y	Y	Y	Y	Y
**P. Farzanegi**	Y	U	Y	U	U	Y	Y	Y	Y	Y	Y	Y	Y
**Yan Zhao**	Y	U	Y	U	U	Y	Y	Y	Y	Y	Y	Y	Y
**Smita DuttaRoy**	Y	U	Y	U	U	Y	Y	Y	Y	Y	Y	Y	Y
**Ryosuke Shimizu**	Y	U	Y	U	U	Y	Y	Y	Y	Y	Y	Y	Y
**Kieran J. Marston**	Y	Y	Y	U	U	Y	Y	Y	Y	Y	Y	Y	Y
**A saBeijer**	Y	U	Y	U	U	Y	Y	Y	Y	Y	Y	Y	Y
**Anna Pedrinolla**	Y	Y	Y	U	U	Y	Y	Y	Y	Y	Y	Y	Y
**Chia-Liang Tsai**	Y	U	Y	U	U	Y	Y	Y	Y	Y	Y	Y	Y
**Su-Youn Cho**	Y	U	Y	U	U	Y	Y	Y	Y	Y	Y	Y	Y
**Su-Youn Cho**	Y	U	Y	U	U	Y	Y	Y	Y	Y	Y	Y	Y

**Sandra Erbs**	Y	Y	Y	U	U	Y	Y	Y	Y	Y	Y	Y	Y
**Kim M. Huffman**	Y	U	Y	U	U	Y	Y	Y	Y	Y	Y	Y	Y
**Xueqiao Jiao**	Y	Y	Y	U	U	Y	Y	Y	Y	Y	Y	Y	Y
**Jesper Krogh**	Y	Y	Y	U	U	Y	Y	Y	Y	Y	Y	Y	Y
**Song LIN**	Y	U	Y	U	U	Y	Y	Y	Y	Y	Y	Y	Y
**Xiaoli Liu**	Y	U	Y	U	U	Y	Y	Y	Y	Y	Y	Y	Y
**Anne Maass**	Y	Y	Y	Y	U	Y	Y	Y	Y				
**Knut Mai**	Y	Y	Y	Y	U	Y	Y	Y	Y	Y	Y	Y	Y
**Stefan Peter Mortensen**	Y	Y	Y	U	U	Y	Y	Y	Y	Y	Y	Y	Y
**Fatih Soke**	Y	Y	Y	U	U	Y	Y	Y	Y	Y	Y	Y	Y
**Mona Mohamed Taha**	Y	U	Y	U	U	Y	Y	Y	Y	Y	Y	Y	Y

Randomized Controlled Trial (RCT) - 13 Items:1. Was true randomization used for assignment of participants to treatment groups?2. Was allocation to treatment groups concealed?3. Were treatment groups similar at the baseline?4. Were participants blind to treatment assignment?5. Were those delivering treatment blind to treatment assignment?6. Were outcomes assessors blind to treatment assignment?7. Were treatment groups treated identically other than the intervention of interest?8. Was follow up complete and if not, were differences between groups in terms of their follow up adequately described and analyzed?9. Were participants analyzed in the groups to which they were randomized?10. Were outcomes measured in the same way for treatment groups?11. Were outcomes measured in a reliable way?12. Was appropriate statistical analysis used?13. Was the trial design appropriate, and any deviations from the standard RCT design (individual randomization, parallel groups) accounted for in the conduct and analysis of the trial?

Quasi-Experimental Study - 9 Items:

1.Is it clear in the study what is the “cause” and what is the “effect” (i.e. there is no confusion about which variable comes first)?2.Was there a control group?3. Were participants included in any comparisons similar?4. Were the participants included in any comparisons receiving similar treatment/care, other than the exposure or intervention of interest?5. Were there multiple measurements of the outcome, both pre and post the intervention/exposure?6. Were the outcomes of participants included in any comparisons measured in the same way?7. Were outcomes measured in a reliable way?8. Was follow-up complete and if not, were differences between groups in terms of their follow-up adequately described and analyzed?9. Was appropriate statistical analysis used

Supplementary S4 — GRADE Assessment for Network Evidence

Table S4.1 GRADE Assessment for Healthy Population

Comparison	Number of studies	Within-study bias	Reporting bias	Indirectness	Imprecision	Heterogeneity	Incoherence	Confidence rating
**AEWR:CG**	1	Some concerns	Low risk	No concerns	Major concerns	No concerns	No concerns	Moderate
**CG:HAE**	1	No concerns	Low risk	No concerns	Major concerns	No concerns	No concerns	Low
**CG:LBFRT**	2	Some concerns	Low risk	No concerns	No concerns	Some concerns	No concerns	Low
**CG:LRT**	1	Some concerns	Low risk	No concerns	No concerns	Major concerns	No concerns	Low
**CG:RT**	5	Some concerns	Low risk	No concerns	Some concerns	Some concerns	No concerns	Very Low
**CG:Taekwondo**	2	Some concerns	Low risk	No concerns	Major concerns	No concerns	No concerns	Very Low
**CG:URT**	1	Some concerns	Low risk	No concerns	No concerns	Major concerns	No concerns	Low
**HBFRT:URT**	1	Some concerns	Low risk	No concerns	Major concerns	No concerns	No concerns	Very Low
**LBFRT:RT**	1	Some concerns	Low risk	No concerns	No concerns	Major concerns	No concerns	Very Low
**LBFRT:URT**	1	Some concerns	Low risk	No concerns	Major concerns	No concerns	No concerns	Very Low
**LRT:RTWV**	1	Some concerns	Low risk	No concerns	Major concerns	No concerns	No concerns	Very Low
**RT:RTH**	1	Some concerns	Low risk	No concerns	Major concerns	No concerns	No concerns	Very Low
**AEWR:HAE**	0	Some concerns	Low risk	No concerns	Major concerns	No concerns	No concerns	Very Low
**AEWR:HBFRT**	0	Some concerns	Low risk	No concerns	Major concerns	No concerns	No concerns	Very Low
**AEWR:LBFRT**	0	Some concerns	Low risk	No concerns	No concerns	Some concerns	No concerns	Very Low
**AEWR:LRT**	0	Some concerns	Low risk	No concerns	No concerns	Major concerns	No concerns	Very Low
**AEWR:RT**	0	Some concerns	Low risk	No concerns	Major concerns	No concerns	No concerns	Very Low
**AEWR:RTH**	0	Some concerns	Low risk	No concerns	Major concerns	No concerns	No concerns	Very Low
**AEWR:RTWV**	0	Some concerns	Low risk	No concerns	Some concerns	Some concerns	No concerns	Very Low
**AEWR:Taekwondo**	0	Some concerns	Low risk	No concerns	Major concerns	No concerns	No concerns	Very Low
**AEWR:URT**	0	Some concerns	Low risk	No concerns	No concerns	Major concerns	No concerns	Very Low

**CG:HBFRT**	0	Some concerns	Low risk	No concerns	Major concerns	No concerns	No concerns	Very Low
**CG:RTH**	0	Some concerns	Low risk	No concerns	Major concerns	No concerns	No concerns	Very Low
**CG:RTWV**	0	Some concerns	Low risk	No concerns	Major concerns	No concerns	No concerns	Very Low
**HAE:HBFRT**	0	Some concerns	Low risk	No concerns	Major concerns	No concerns	No concerns	Very Low
**HAE:LBFRT**	0	Some concerns	Low risk	No concerns	Some concerns	Some concerns	No concerns	Very Low
**HAE:LRT**	0	Some concerns	Low risk	No concerns	Major concerns	No concerns	No concerns	Very Low
**HAE:RT**	0	No concerns	Low risk	No concerns	Major concerns	No concerns	No concerns	Very Low
**HAE:RTH**	0	Some concerns	Low risk	No concerns	Major concerns	No concerns	No concerns	Very Low
**HAE:RTWV**	0	Some concerns	Low risk	No concerns	Major concerns	No concerns	No concerns	Very Low
**HAE:Taekwondo**	0	Some concerns	Low risk	No concerns	Major concerns	No concerns	No concerns	Very Low
**HAE:URT**	0	Some concerns	Low risk	No concerns	Major concerns	No concerns	No concerns	Very Low
**HBFRT:LBFRT**	0	Some concerns	Low risk	No concerns	Major concerns	No concerns	No concerns	Very Low
**HBFRT:LRT**	0	Some concerns	Low risk	No concerns	Major concerns	No concerns	No concerns	Very Low
**HBFRT:RT**	0	Some concerns	Low risk	No concerns	Major concerns	No concerns	No concerns	Very Low
**HBFRT:RTH**	0	Some concerns	Low risk	No concerns	Major concerns	No concerns	No concerns	Very Low
**HBFRT:RTWV**	0	Some concerns	Low risk	No concerns	Major concerns	No concerns	No concerns	Very Low
**HBFRT:Taekwondo**	0	Some concerns	Low risk	No concerns	Major concerns	No concerns	No concerns	Very Low
**LBFRT:LRT**	0	Some concerns	Low risk	No concerns	Major concerns	No concerns	No concerns	Very Low
**LBFRT:RTH**	0	Some concerns	Low risk	No concerns	Major concerns	No concerns	No concerns	Very Low
**LBFRT:RTWV**	0	Some concerns	Low risk	No concerns	Major concerns	No concerns	No concerns	Very Low
**LBFRT:Taekwondo**	0	Some concerns	Low risk	No concerns	Some concerns	Some concerns	No concerns	Very Low
**LRT:RT**	0	Some concerns	Low risk	No concerns	Major concerns	No concerns	No concerns	Very Low
**LRT:RTH**	0	Some concerns	Low risk	No concerns	Major concerns	No concerns	No concerns	Very Low
**LRT:Taekwondo**	0	Some concerns	Low risk	No concerns	Major concerns	No concerns	No concerns	Very Low
**LRT:URT**	0	Some concerns	Low risk	No concerns	Major concerns	No concerns	No concerns	Very Low

**RT:RTWV**	0	Some concerns	Low risk	No concerns	Major concerns	No concerns	No concerns	Very Low
**RT:Taekwondo**	0	Some concerns	Low risk	No concerns	Major concerns	No concerns	No concerns	Very Low
**RT:URT**	0	Some concerns	Low risk	No concerns	Major concerns	No concerns	No concerns	Very Low
**RTH:RTWV**	0	Some concerns	Low risk	No concerns	Major concerns	No concerns	No concerns	Very Low
**RTH:Taekwondo**	0	Some concerns	Low risk	No concerns	Major concerns	No concerns	No concerns	Very Low
**RTH:URT**	0	Some concerns	Low risk	No concerns	Major concerns	No concerns	No concerns	Very Low
**RTWV:Taekwondo**	0	Some concerns	Low risk	No concerns	Major concerns	No concerns	No concerns	Very Low
**RTWV:URT**	0	Some concerns	Low risk	No concerns	Major concerns	No concerns	No concerns	Very Low
**Taekwondo:URT**	0	Some concerns	Low risk	No concerns	Major concerns	No concerns	No concerns	Very Low

Table S4.2 GRADE Assessment for Healthy Population

Comparison	Number of studies	Within-study bias	Reporting bias	Indirectness	Imprecision	Heterogeneity	Incoherence	Confidence rating
**AEH:HAE**	1	No concerns	Low risk	No concerns	Major concerns	No concerns	No concerns	Low
**AEWR:AWRH**	1	No concerns	Low risk	No concerns	Major concerns	No concerns	No concerns	Low
**AEWR:MAE**	1	Some concerns	Low risk	No concerns	Major concerns	No concerns	No concerns	Very Low
**CG:HAE**	2	Some concerns	Low risk	No concerns	Major concerns	No concerns	No concerns	Very Low
**CG:MAE**	1	Some concerns	Low risk	No concerns	Major concerns	No concerns	No concerns	Very Low
**CG:RT**	1	No concerns	Low risk	No concerns	Major concerns	No concerns	No concerns	Low
**HAE:RIPC**	1	No concerns	Low risk	No concerns	Major concerns	No concerns	No concerns	Low
**AEH:AEWR**	0	Some concerns	Low risk	No concerns	Major concerns	No concerns	No concerns	Very Low
**AEH:AWRH**	0	Some concerns	Low risk	No concerns	Major concerns	No concerns	No concerns	Very Low
**AEH:CG**	0	Some concerns	Low risk	No concerns	Major concerns	No concerns	No concerns	Very Low
**AEH:MAE**	0	Some concerns	Low risk	No concerns	Major concerns	No concerns	No concerns	Very Low
**AEH:RIPC**	0	No concerns	Low risk	No concerns	Major concerns	No concerns	No concerns	Low
**AEH:RT**	0	No concerns	Low risk	No concerns	Major concerns	No concerns	No concerns	Low

**AEWR:CG**	0	Some concerns	Low risk	No concerns	Major concerns	No concerns	No concerns	Very Low
**AEWR:HAE**	0	Some concerns	Low risk	No concerns	Major concerns	No concerns	No concerns	Very Low
**AEWR:RIPC**	0	Some concerns	Low risk	No concerns	Major concerns	No concerns	No concerns	Very Low
**AEWR:RT**	0	Some concerns	Low risk	No concerns	Major concerns	No concerns	No concerns	Very Low
**AWRH:CG**	0	Some concerns	Low risk	No concerns	Major concerns	No concerns	No concerns	Very Low
**AWRH:HAE**	0	Some concerns	Low risk	No concerns	Major concerns	No concerns	No concerns	Very Low
**AWRH:MAE**	0	Some concerns	Low risk	No concerns	Major concerns	No concerns	No concerns	Very Low
**AWRH:RIPC**	0	Some concerns	Low risk	No concerns	Major concerns	No concerns	No concerns	Very Low
**AWRH:RT**	0	Some concerns	Low risk	No concerns	Major concerns	No concerns	No concerns	Very Low
**CG:RIPC**	0	Some concerns	Low risk	No concerns	Major concerns	No concerns	No concerns	Very Low
**HAE:MAE**	0	Some concerns	Low risk	No concerns	Major concerns	No concerns	No concerns	Very Low
**HAE:RT**	0	Some concerns	Low risk	No concerns	Major concerns	No concerns	No concerns	Very Low
**MAE:RIPC**	0	Some concerns	Low risk	No concerns	Major concerns	No concerns	No concerns	Very Low
**MAE:RT**	0	Some concerns	Low risk	No concerns	Major concerns	No concerns	No concerns	Very Low
**RIPC:RT**	0	No concerns	Low risk	No concerns	Major concerns	No concerns	No concerns	Low

Table S4.3 GRADE Assessment for Healthy Population

Comparison	Number of studies	Within-study bias	Reporting bias	Indirectness	Imprecision	Heterogeneity	Incoherence	Confidence rating
**AEH:MAE**	1	No concerns	Low risk	No concerns	Major concerns	No concerns	No concerns	Low
**AETOT:HAE**	1	No concerns	Low risk	No concerns	Major concerns	No concerns	No concerns	Low
**AEWR:CG**	2	Some concerns	Low risk	No concerns	Major concerns	No concerns	No concerns	Very Low
**CG:HAE**	1	No concerns	Low risk	No concerns	Major concerns	No concerns	No concerns	Low
**CG:HIIT**	1	Some concerns	Low risk	No concerns	Major concerns	No concerns	No concerns	Very Low
**CG:MAE**	3	Some concerns	Low risk	No concerns	Major concerns	No concerns	No concerns	Very Low
**CG:RT**	2	Some concerns	Low risk	No concerns	Major concerns	No concerns	No concerns	Very Low

**CG:TWR**	1	Some concerns	Low risk	No concerns	Major concerns	No concerns	No concerns	Very Low
**CG:Taiji**	1	Some concerns	Low risk	No concerns	Major concerns	No concerns	No concerns	Very Low
**CG:URT**	1	Some concerns	Low risk	No concerns	Major concerns	No concerns	No concerns	Very Low
**HIIT:MAE**	1	No concerns	Low risk	No concerns	Major concerns	No concerns	No concerns	Low
**AEH:AETOT**	0	No concerns	Low risk	No concerns	Major concerns	No concerns	No concerns	Low
**AEH:AEWR**	0	No concerns	Low risk	No concerns	Major concerns	No concerns	No concerns	Low
**AEH:CG**	0	No concerns	Low risk	No concerns	Major concerns	No concerns	No concerns	Low
**AEH:HAE**	0	No concerns	Low risk	No concerns	Major concerns	No concerns	No concerns	Low
**AEH:HIIT**	0	No concerns	Low risk	No concerns	Major concerns	No concerns	No concerns	Low
**AEH:RT**	0	Some concerns	Low risk	No concerns	Major concerns	No concerns	No concerns	Very Low
**AEH:TWR**	0	Some concerns	Low risk	No concerns	Major concerns	No concerns	No concerns	Very Low
**AEH:Taiji**	0	Some concerns	Low risk	No concerns	Major concerns	No concerns	No concerns	Very Low
**AEH:URT**	0	Some concerns	Low risk	No concerns	Major concerns	No concerns	No concerns	Very Low
**AETOT:AEWR**	0	No concerns	Low risk	No concerns	Major concerns	No concerns	No concerns	Low
**AETOT:CG**	0	No concerns	Low risk	No concerns	Major concerns	No concerns	No concerns	Low
**AETOT:HIIT**	0	No concerns	Low risk	No concerns	Major concerns	No concerns	No concerns	Low
**AETOT:MAE**	0	No concerns	Low risk	No concerns	Major concerns	No concerns	No concerns	Low
**AETOT:RT**	0	No concerns	Low risk	No concerns	Major concerns	No concerns	No concerns	Low
**AETOT:TWR**	0	No concerns	Low risk	No concerns	Major concerns	No concerns	No concerns	Low
**AETOT:Taiji**	0	No concerns	Low risk	No concerns	Major concerns	No concerns	No concerns	Low
**AETOT:URT**	0	No concerns	Low risk	No concerns	Major concerns	No concerns	No concerns	Low
**AEWR:HAE**	0	No concerns	Low risk	No concerns	Major concerns	No concerns	No concerns	Low
**AEWR:HIIT**	0	Some concerns	Low risk	No concerns	Major concerns	No concerns	No concerns	Very Low
**AEWR:MAE**	0	Some concerns	Low risk	No concerns	Major concerns	No concerns	No concerns	Very Low
**AEWR:RT**	0	Some concerns	Low risk	No concerns	Major concerns	No concerns	No concerns	Very Low

**AEWR:TWR**	0	Some concerns	Low risk	No concerns	Major concerns	No concerns	No concerns	Very Low
**AEWR:Taiji**	0	Some concerns	Low risk	No concerns	Major concerns	No concerns	No concerns	Very Low
**AEWR:URT**	0	Some concerns	Low risk	No concerns	Major concerns	No concerns	No concerns	Very Low
**HAE:HIIT**	0	No concerns	Low risk	No concerns	Major concerns	No concerns	No concerns	Low
**HAE:MAE**	0	No concerns	Low risk	No concerns	Major concerns	No concerns	No concerns	Low
**HAE:RT**	0	Some concerns	Low risk	No concerns	Major concerns	No concerns	No concerns	Low
**HAE:TWR**	0	Some concerns	Low risk	No concerns	Major concerns	No concerns	No concerns	Very Low
**HAE:Taiji**	0	Some concerns	Low risk	No concerns	Major concerns	No concerns	No concerns	Very Low
**HAE:URT**	0	Some concerns	Low risk	No concerns	Major concerns	No concerns	No concerns	Very Low
**HIIT:RT**	0	Some concerns	Low risk	No concerns	Major concerns	No concerns	No concerns	Very Low
**HIIT:TWR**	0	Some concerns	Low risk	No concerns	Major concerns	No concerns	No concerns	Very Low
**HIIT:Taiji**	0	Some concerns	Low risk	No concerns	Major concerns	No concerns	No concerns	Very Low
**HIIT:URT**	0	Some concerns	Low risk	No concerns	Major concerns	No concerns	No concerns	Very Low
**MAE:RT**	0	Some concerns	Low risk	No concerns	Major concerns	No concerns	No concerns	Very Low
**MAE:TWR**	0	Some concerns	Low risk	No concerns	Major concerns	No concerns	No concerns	Very Low
**MAE:Taiji**	0	Some concerns	Low risk	No concerns	Major concerns	No concerns	No concerns	Very Low
**MAE:URT**	0	Some concerns	Low risk	No concerns	Major concerns	No concerns	No concerns	Very Low
**RT:TWR**	0	Some concerns	Low risk	No concerns	Major concerns	No concerns	No concerns	Very Low
**RT:Taiji**	0	Some concerns	Low risk	No concerns	Major concerns	No concerns	No concerns	Very Low
**RT:URT**	0	Some concerns	Low risk	No concerns	Major concerns	No concerns	No concerns	Very Low
**Taiji:TWR**	0	Some concerns	Low risk	No concerns	Major concerns	No concerns	No concerns	Very Low
**TWR:URT**	0	Some concerns	Low risk	No concerns	Major concerns	No concerns	No concerns	Very Low
**Taiji:URT**	0	Some concerns	Low risk	No concerns	Major concerns	No concerns	No concerns	Very Low

Supplementary S5 — Global Inconsistency Test

Table S5. Inconsistency test results for network meta-analysis

Outcome	Null hypothesis (example)	χ^2 (df)	p-value	Conclusion
**Health**	des_EG = 0; des_EK = 0	0.45 (2)	0.797	consistency
**Obesity**	-cons(E) + cons(G) = 0	0.29 (1)	0.592	consistency
**Disease**	des_DF = 0	0.00 (1)	0.954	consistency

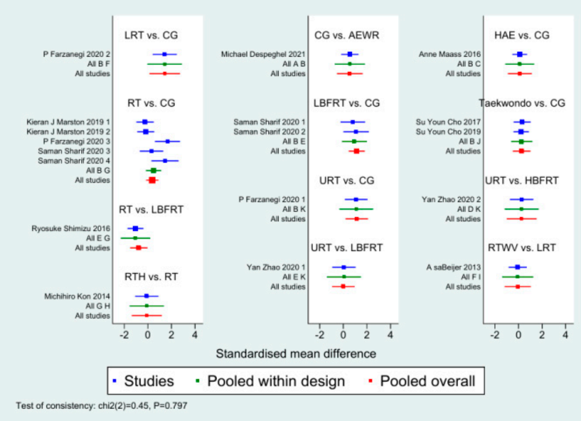

Figure S5.1. Consistency analysis of healthy population.

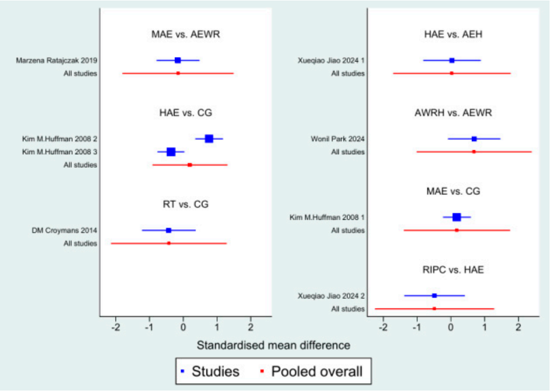

Figure S5.2. Consistency analysis of obese population.

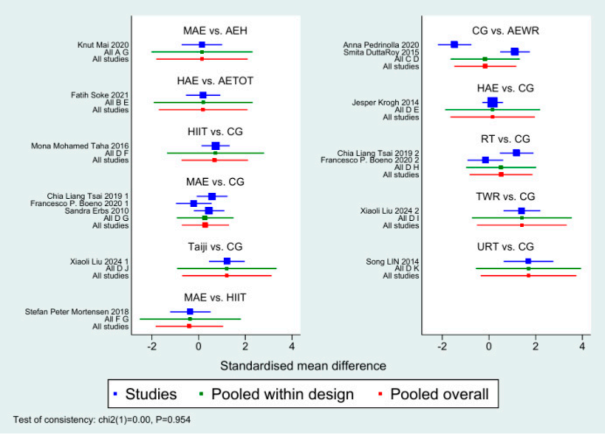

Figure S5.3. Consistency analysis of patients

Supplemental Table S6 Node splitting method-------local inconsistency

S	id		Direct		Indirect		Difference		
			Coef.	Std. Err.	Coef.	Std. Err.	Coef.	Std. Err.	P>|z|
**Healthy population**									
**AEWR**	CG	*	0.5505032	0.5522123	-0.4893813	76.4151	1.039884	76.4171	0.989
**CG**	HAE	*	0.1095495	0.5175347	-1.106396	642.2437	1.215945	642.2439	0.998
**CG**	LBFRT		0.9373111	0.50231	1.366817	0.5365994	-0.4295059	0.7349463	0.559
**CG**	LRT	*	1.434141	0.6593949	-0.6348244	282.9486	2.068965	282.9493	0.994
**CG**	RT		0.467969	0.3046147	-0.0877445	0.7299563	0.5557135	0.7909328	0.482
**CG**	Taekwondo	*	0.2714515	0.3801192	-1.10493	451.836	1.376382	451.8361	0.998
**CG**	URT		1.092956	0.682839	1.206521	0.7993141	-0.1135648	1.051272	0.914
**HBFRT**	URT	*	0.2692808	0.6485672	3.382565	632.6376	-3.113284	632.638	0.996
**LBFRT**	RT		-1.047463	0.5775752	-0.4917635	0.540359	-0.5556992	0.7909368	0.482
**LBFRT**	URT		0.0515719	0.6908355	-0.062026	0.7924158	0.1135979	1.051274	0.914
**LRT**	RTWV	*	-0.0643039	0.5668906	-3.969187	626.5481	3.904883	626.5483	0.995
**RT**	RTH	*	-0.087849	0.6494092	-1.837096	630.5329	1.749247	630.5333	0.998
**Patients**									
**AEH**	MAE	*	0.1434596	0.9966232	-0.0093563	84.41352	0.1528159	84.41941	0.999
**AETOT**	HAE	*	0.1948252	0.9680247	0.0054724	632.6787	0.1893528	632.6794	1
**AEWR**	CG	*	-0.1573192	0.6773943	-0.3155631	447.2397	0.1582439	447.2402	1
**CG**	HAE	*	0.1554702	0.9214491	0.2586101	282.9896	-0.1031399	282.9911	1
**CG**	HITT		0.7281588	1.057157	0.6334944	1.265707	0.0946644	1.64912	0.954
**CG**	MAE		0.2783438	0.6188939	0.3730353	1.528361	-0.0946915	1.648914	0.954
**CG**	RT	*	0.520241	0.6856421	0.3004958	450.3276	0.2197451	450.3281	1

**CG**	TWR	*	1.408508	0.9795831	0.2982711	634.6224	1.110237	634.6231	0.999
**CG**	Taiji	*	1.209278	0.9746877	0.2985795	636.241	0.9106986	636.2418	0.999
**CG**	URT	*	1.693778	1.04507	0.2983047	632.4487	1.395473	632.4496	0.998
**HITT**	MAE		-0.355147	1.104076	-0.4498037	1.22499	0.0946566	1.649116	0.954

Supplementary S7 — Network Plots for All Outcomes

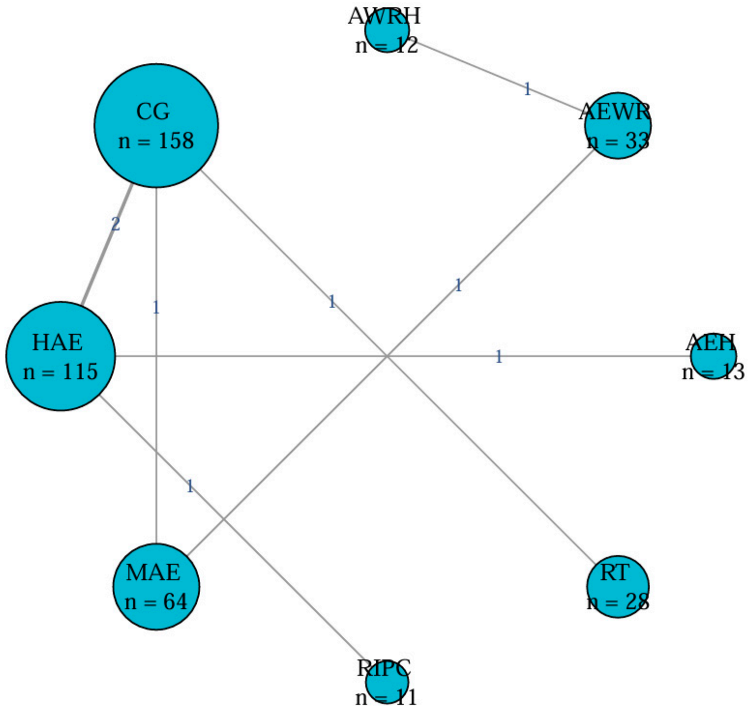

Figure S7.1 Network plot for Obese population

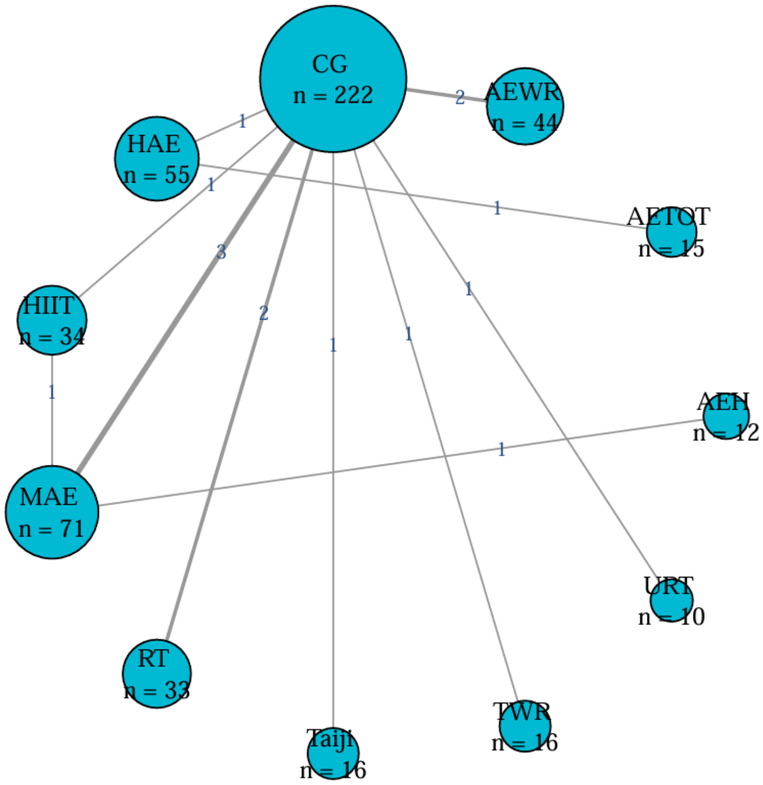

Figure S7.2 Network plot for Patients

Supplemental S8 League table

Table S8.1 League Table of Healthy population

URT										
0.87 (-0.34,2.08)	Taekwondo									
-0.23 (-2.18,1.72)	-1.10 (-2.96,0.76)	RTWV								
0.86 (-0.79,2.50)	-0.01 (-1.58,1.55)	1.08 (-1.11,3.27)	RTH							
0.77 (-0.27,1.81)	-0.10 (-1.01,0.81)	1.00 (-0.79,2.78)	-0.09 (-1.36,1.18)	RT						
-0.29 (-1.90,1.31)	-1.16 (-2.65,0.33)	-0.06 (-1.18,1.05)	-1.15 (-3.04,0.74)	-1.06 (-2.45,0.33)	LRT					
0.00 (-0.95,0.96)	-0.87 (-1.87,0.14)	0.23 (-1.60,2.07)	-0.85 (-2.32,0.62)	-0.76 (-1.50,-0.03)	0.30 (-1.16,1.75)	LBFRT				
0.27 (-1.00,1.54)	-0.60 (-2.35,1.15)	0.50 (-1.83,2.83)	-0.59 (-2.66,1.49)	-0.50 (-2.14,1.14)	0.56 (-1.48,2.61)	0.27 (-1.32,1.86)	HBFRT			
1.03 (-0.36,2.42)	0.16 (-1.10,1.42)	1.26 (-0.72,3.24)	0.18 (-1.53,1.89)	0.26 (-0.88,1.41)	1.32 (-0.32,2.97)	1.03 (-0.19,2.25)	0.76 (-1.12,2.65)	HAE		
1.14 (0.19,2.09)	0.27 (-0.47,1.02)	1.37 (-0.33,3.07)	0.29 (-1.09,1.66)	0.37 (-0.15,0.90)	1.43 (0.14,2.73)	1.14 (0.46,1.82)	0.87 (-0.72,2.46)	0.11 (-0.90,1.12)	CG	
1.69 (0.25,3.13)	0.82 (-0.49,2.14)	1.92 (-0.10,3.94)	0.84 (-0.91,2.59)	0.92 (-0.28,2.13)	1.98 (0.30,3.67)	1.69 (0.41,2.97)	1.42 (-0.50,3.34)	0.66 (-0.82,2.14)	0.55 (-0.53,1.63)	AEWR

Table S8.2 League Table of Obese population

RT							
-0.14 (-2.84,2.55)	RIPC						
-0.61 (-2.93,1.72)	-0.46 (-3.07,2.15)	MAE					
-0.63 (-2.67,1.41)	-0.48 (-2.24,1.28)	-0.02 (-1.94,1.90)	HAE				
-0.43 (-2.14,1.28)	-0.28 (-2.36,1.80)	0.18 (-1.39,1.75)	0.20 (-0.91,1.31)	CG			
-1.45 (-4.77,1.86)	-1.31 (-4.83,2.21)	-0.85 (-3.21,1.52)	-0.83 (-3.88,2.22)	-1.03 (-3.87,1.81)	AWRH		
-0.76 (-3.61,2.08)	-0.62 (-3.70,2.47)	-0.15 (-1.80,1.49)	-0.13 (-2.67,2.40)	-0.34 (-2.61,1.94)	0.69 (-1.01,2.39)	AEWR	
-0.59 (-3.27,2.09)	-0.45 (-2.92,2.03)	0.01 (-2.58,2.61)	0.03 (-1.71,1.77)	-0.17 (-2.23,1.89)	0.86 (-2.65,4.37)	0.17 (-2.90,3.24)	AEH

Table S8.3 League Table of Patients

URT										
0.48 (-2.32,3.29)	Taiji									
0.29 (-2.52,3.09)	-0.20 (-2.91,2.51)	TWR								
1.17 (-1.28,3.62)	0.69 (-1.65,3.02)	0.89 (-1.46,3.23)	RT							
1.40 (-0.88,3.68)	0.92 (-1.24,3.08)	1.12 (-1.05,3.28)	0.23 (-1.45,1.91)	MAE						
1.00 (-1.49,3.50)	0.52 (-1.86,2.90)	0.72 (-1.67,3.11)	-0.17 (-2.13,1.79)	-0.40 (-1.84,1.05)	HIIT					
1.54 (-1.19,4.27)	1.05 (-1.58,3.68)	1.25 (-1.38,3.89)	0.36 (-1.89,2.62)	0.14 (-1.93,2.21)	0.53 (-1.77,2.84)	HAE				
1.69 (-0.35,3.74)	1.21 (-0.70,3.12)	1.41 (-0.51,3.33)	0.52 (-0.82,1.86)	0.29 (-0.72,1.30)	0.69 (-0.74,2.12)	0.16 (-1.65,1.96)	CG			
1.54 (-0.90,3.98)	1.05 (-1.27,3.38)	1.25 (-1.08,3.59)	0.36 (-1.53,2.25)	0.14 (-1.53,1.80)	0.53 (-1.42,2.48)	-0.00 (-2.24,2.24)	-0.16 (-1.48,1.17)	AEWR		
1.73 (-1.59,5.06)	1.25 (-1.99,4.49)	1.45 (-1.80,4.70)	0.56 (-2.38,3.50)	0.33 (-2.47,3.14)	0.73 (-2.25,3.71)	0.19 (-1.70,2.09)	0.04 (-2.58,2.66)	0.20 (-2.74,3.13)	AETOT	
1.54 (-1.46,4.55)	1.06 (-1.85,3.97)	1.26 (-1.66,4.18)	0.37 (-2.21,2.95)	0.14 (-1.81,2.10)	0.54 (-1.89,2.97)	0.01 (-2.84,2.85)	-0.15 (-2.35,2.05)	0.01 (-2.56,2.58)	-0.19 (-3.61,3.23)	AEH

Supplemental S9 Pairwise comparison forest plot

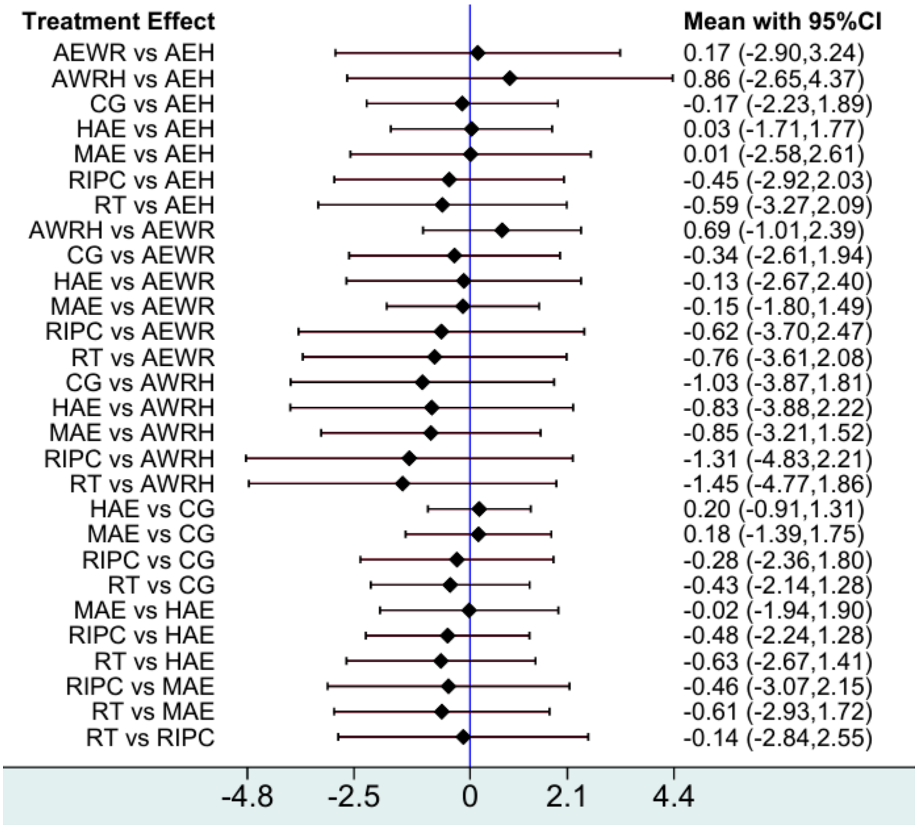

Figure S9.1 Forest Plots of Obese population

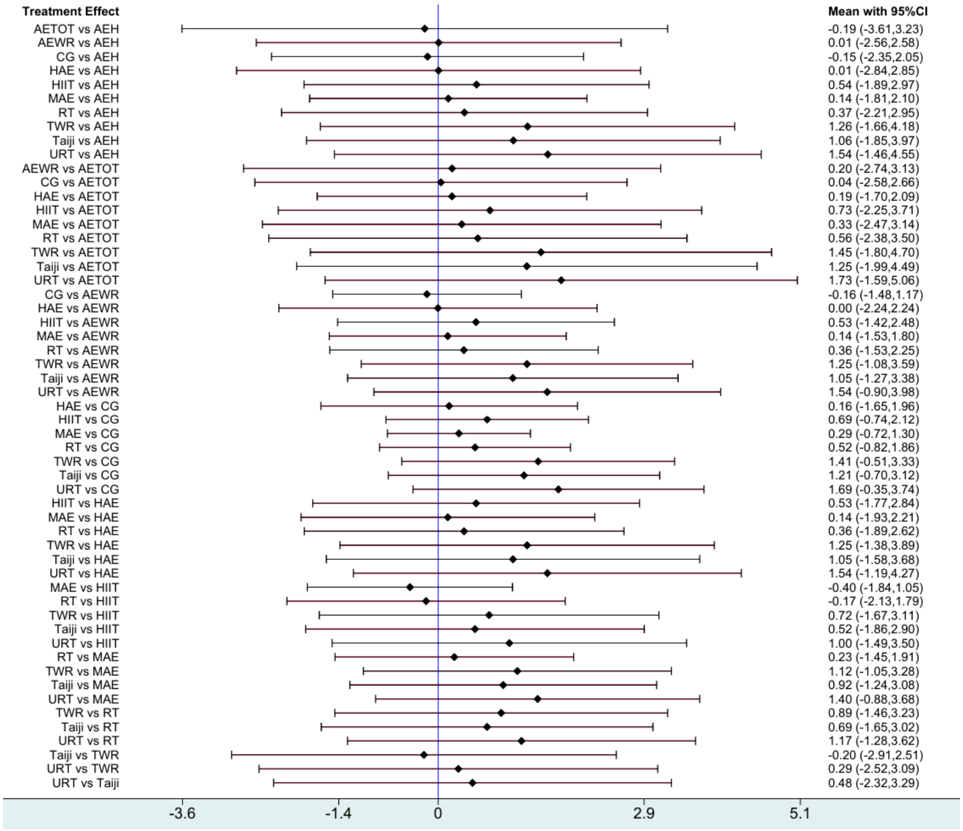

Figure S9.2 Forest Plots of Patients

Supplemental S10 Overall SUCRA Rankings

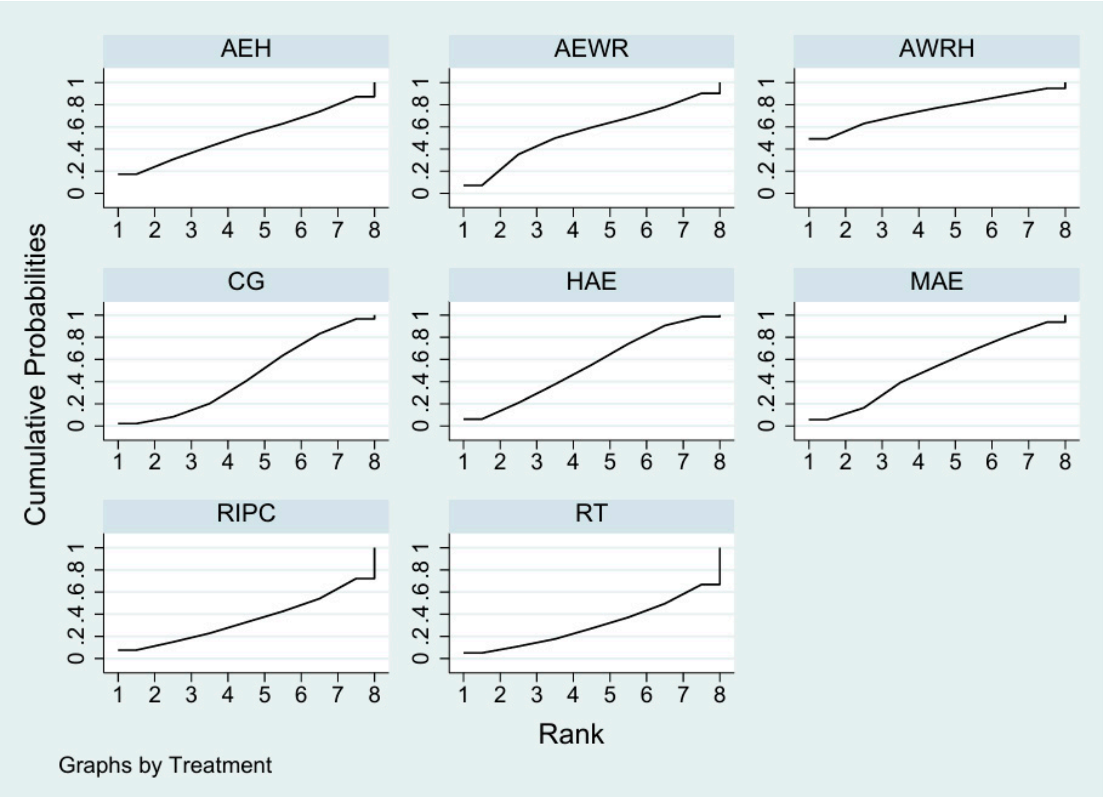

Figure S10.1 Overall SUCRA Rankings for Obese population

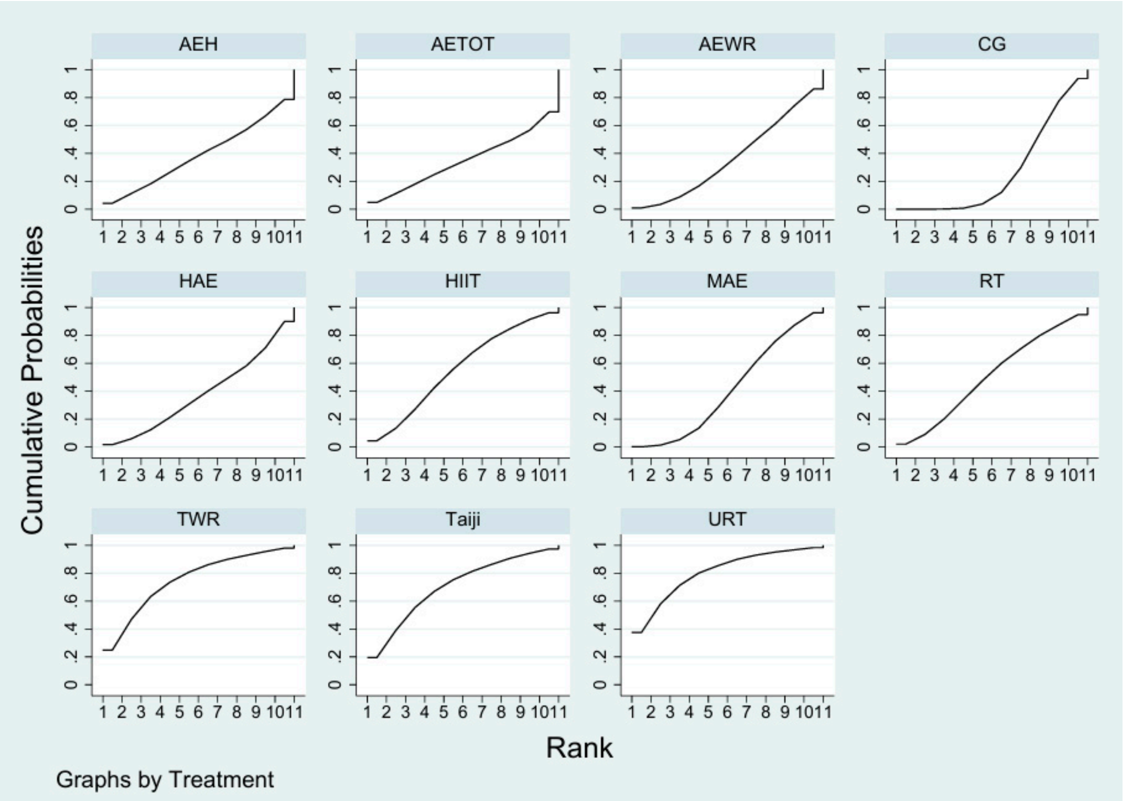

Figure S10.2 Overall SUCRA Rankings for Patients

Supplementary S11 — Funnel Plots for Publication Bias

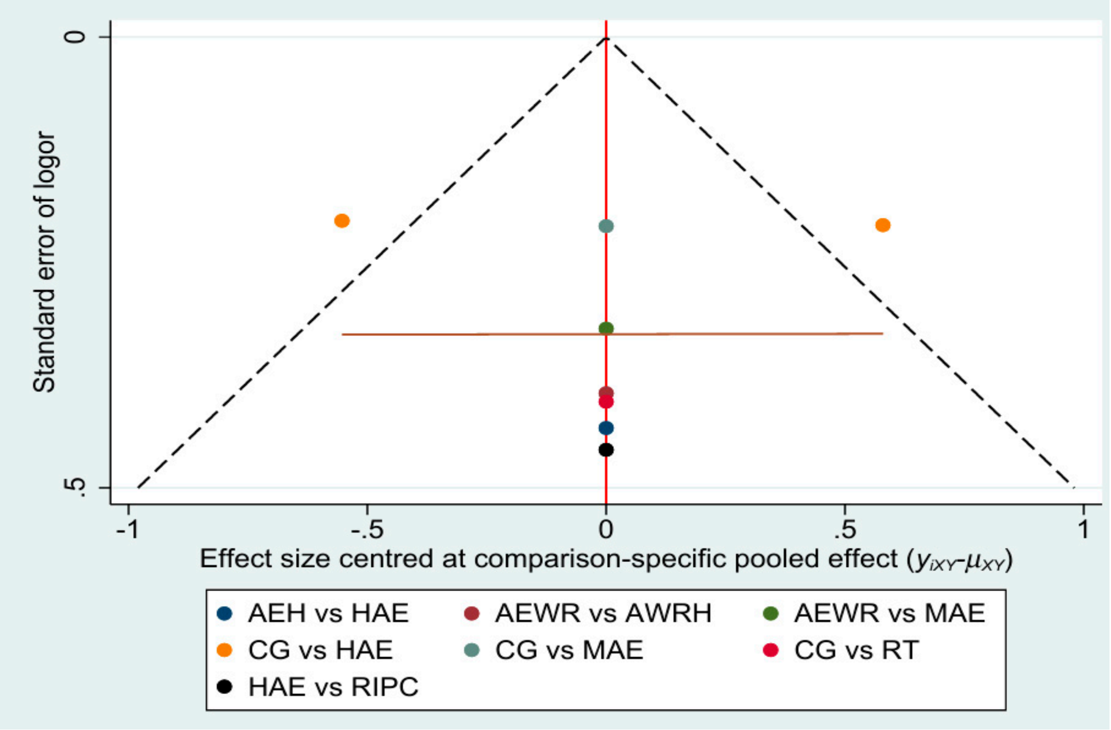

Figure S11.1 Funnel Plots of Obese population

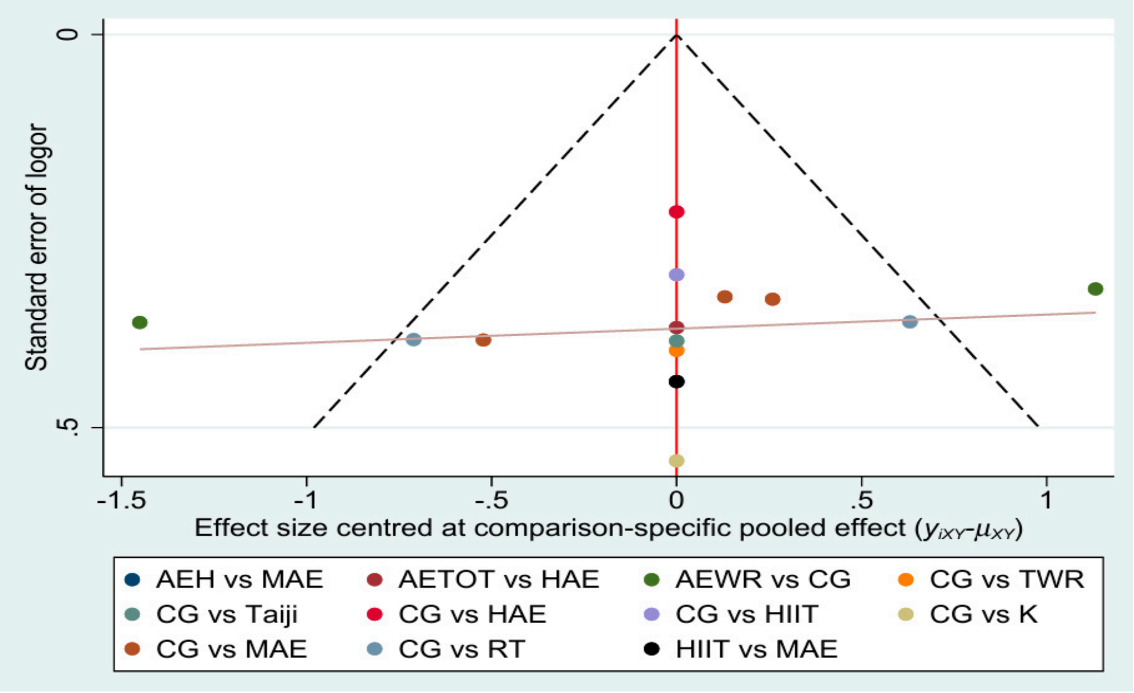

Figure S11.2 Funnel Plots of Patients

Supplementary S12 — Meta-Regression Results

Table S12 Meta-Regression Results

Outcome	Covariate	Beta	SE	P-value
**Healthy population**	AGE	-0.01	0.006758	0.011909
	Male	0.004692	0.004102	0.064492
	Method	0.1538	0.1398	0.2711
	Source	0.307	0.2447	0.2098
**Obese population**	AGE	0.023975	0.013888	0.084294
	Male	-0.0088	0.006208	0.156436
	Method	-0.0596	0.2599	0.8185
	Source	0.1207	0.2455	0.6228
**Patients**	AGE	-0.01	0.016124	0.535078
	Male	0.004692	0.007851	0.550105
	Method	0.4785	0.228	0.0359
	Source	0.3176	0.2917	0.2762

Note. Meta-regression was conducted to explore potential sources of heterogeneity. Age showed a significant effect in the healthy population, while detection method was significant in patients; no other covariates reached statistical significance.

Supplementary S13— Network Meta-Regression Bubble Plots

Healthy population

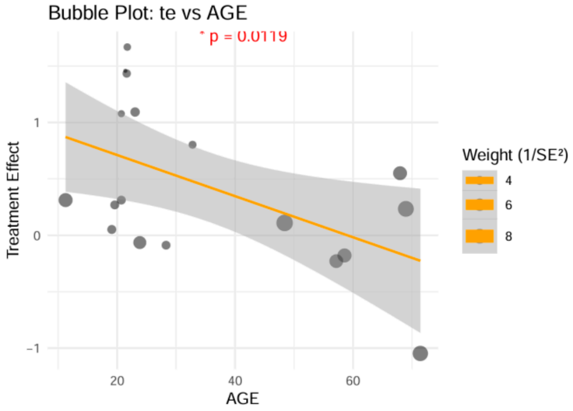

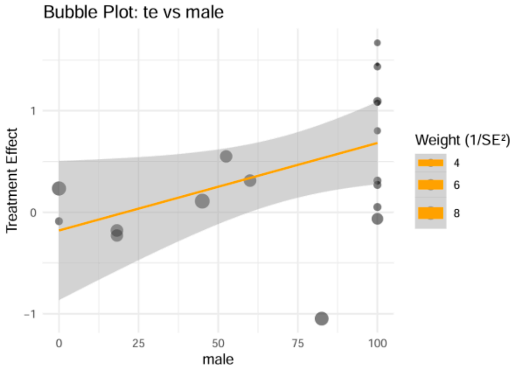

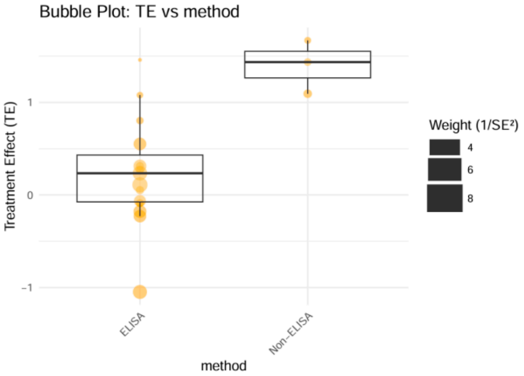

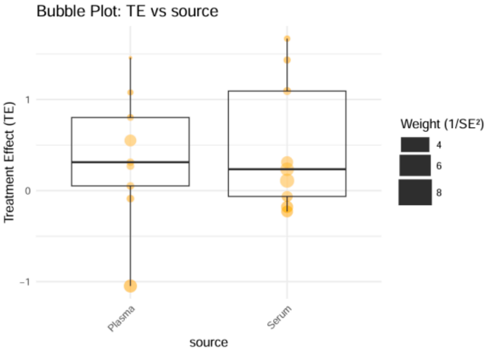

Obese population

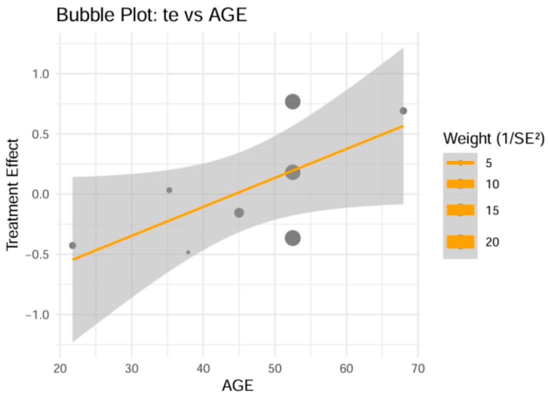
Bubble Plot: te vs AGE
Treatment Effect
AGE
Weight (1/SE²)
5
10
15
20

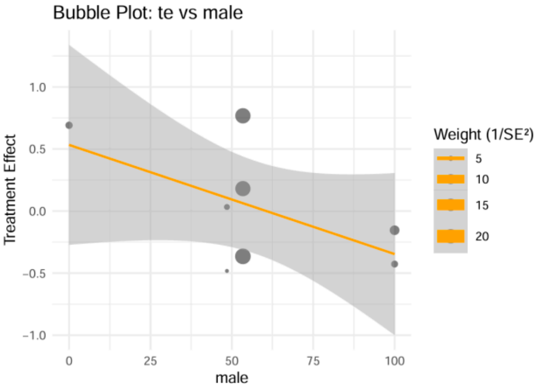
Bubble Plot: te vs male
Treatment Effect
male
Weight (1/SE²)
5
10
15
20

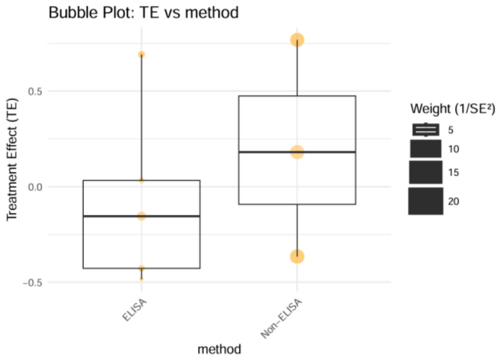
Bubble Plot: TE vs method
Treatment Effect (TE)
ELISA
Non-ELISA
method
Weight (1/SE²)
5
10
15
20

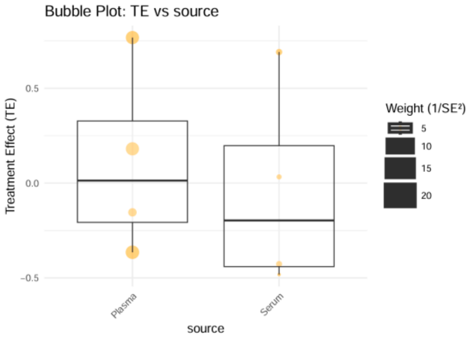
Bubble Plot: TE vs source
Treatment Effect (TE)
Plasma
Serum
source
Weight (1/SE²)
5
10
15
20

Patients

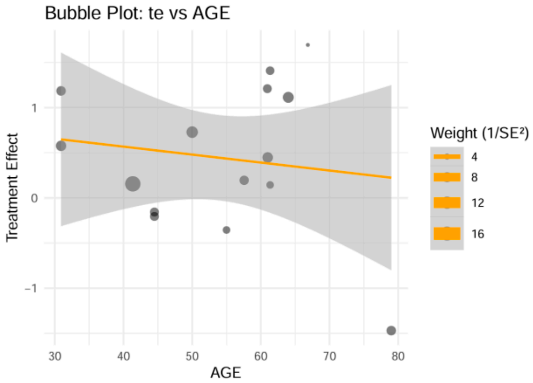
Bubble Plot: te vs AGE
Treatment Effect
AGE
Weight (1/SE²)
4
8
12
16

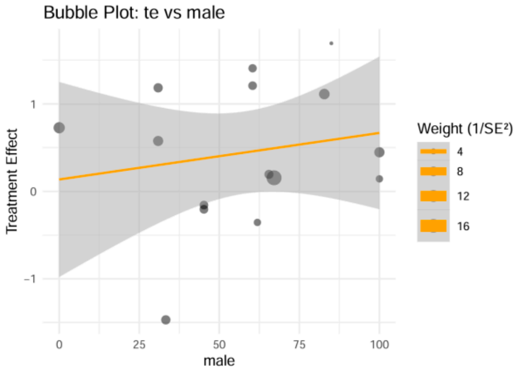
Bubble Plot: te vs male
Treatment Effect
male
Weight (1/SE²)
4
8
12
16

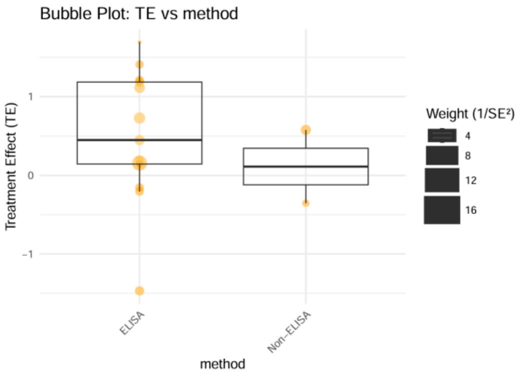
Bubble Plot: TE vs method
Treatment Effect (TE)
1
0
−1
ELISA
Non−ELISA
method
Weight (1/SE²)
4
8
12
16

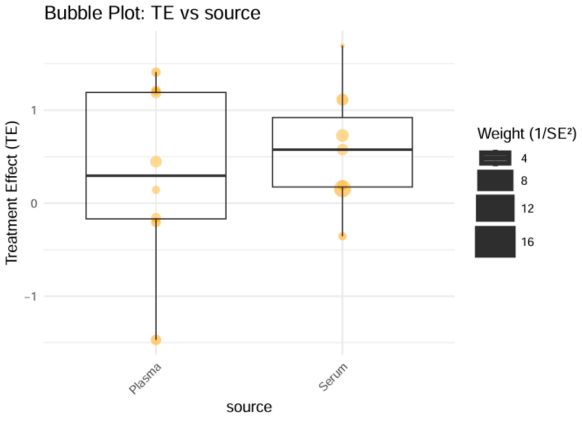
Bubble Plot: TE vs source
Treatment Effect (TE)
1
0
−1
Plasma
Serum
source
Weight (1/SE²)
4
8
12
16

Supplementary S14— Dose-response results

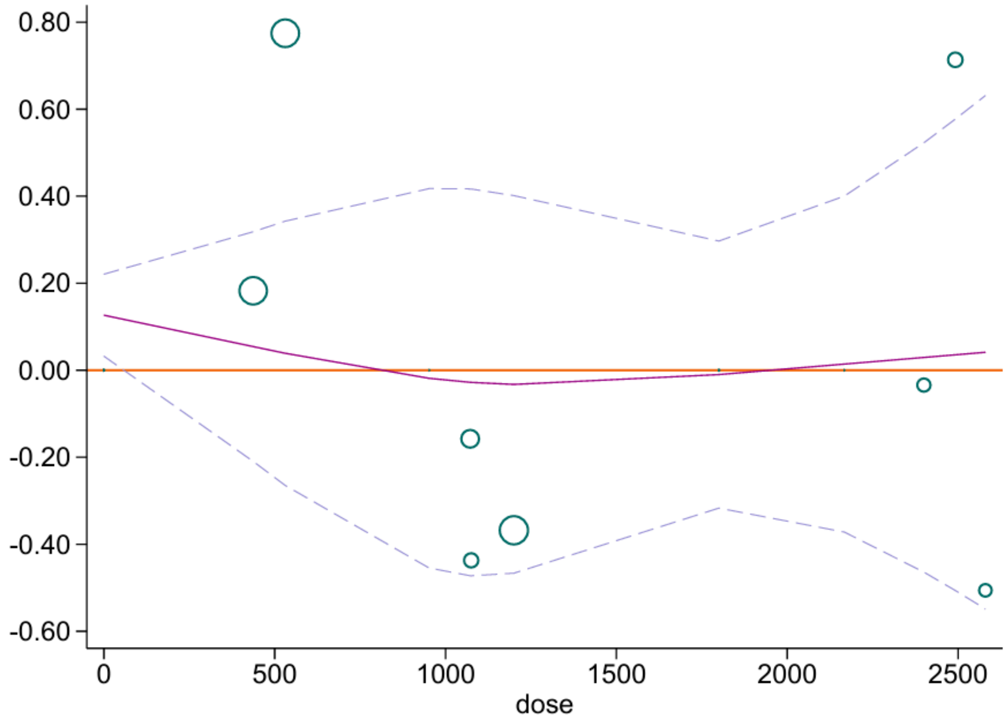

Figure S14.1 Dose-response results of Obese population

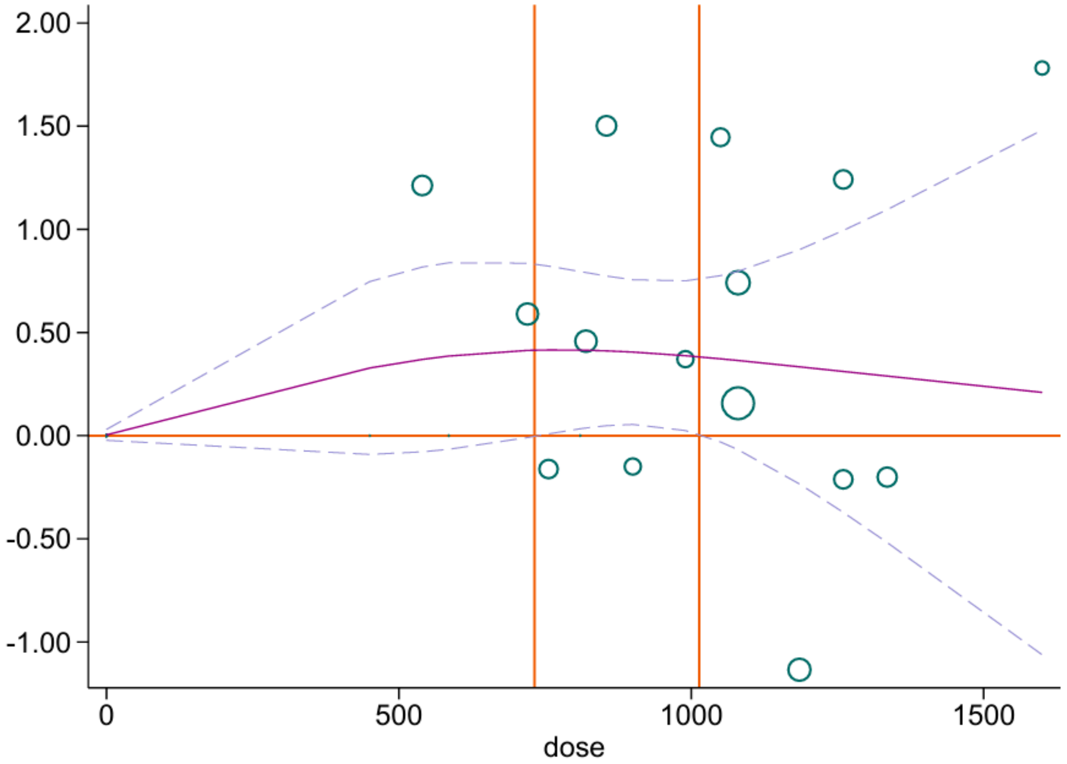

Figure S14.2 Dose-response results of Patients

References:

1. Sharifi S, Monazzami A, Nikousefat Z et al.The acute and chronic effects of resistance training with blood flow restriction on hormonal responses in untrained young men: A comparison of frequency.Cellular and molecular biology (Noisy-le-Grand, France).2020, **66**(1):1-8.
2. Boeno FP, Ramis TR, Munhoz SV et al.Effect of aerobic and resistance exercise training on inflammation, endothelial function and ambulatory blood pressure in middle-aged hypertensive patients.Journal of hypertension.2020, **38**(12):2501-2509. doi:10.1097/hjh.0000000000002581
3. Park W, Park HY, Kim SW.Effects of 12 Weeks of Combined Exercise Training in Normobaric Hypoxia on Arterial Stiffness, Inflammatory Biomarkers, and Red Blood Cell Hemorheological Function in Obese Older Women.Healthcare (Basel, Switzerland).2024, **12**(18). doi:10.3390/healthcare12181887
4. Despeghel M, Reichel T, Zander J et al.Effects of a 6 Week Low-Dose Combined Resistance and Endurance Training on T Cells and Systemic Inflammation in the Elderly.Cells.2021, **10**(4). doi:10.3390/cells10040843
5. Ratajczak M, Skrypnik D, Bogdański P et al.Effects of Endurance and Endurance-Strength Training on Endothelial Function in Women with Obesity: A Randomized Trial.International journal of environmental research and public health.2019, **16**(21). doi:10.3390/ijerph16214291
6. Croymans DM, Krell SL, Oh CS et al.Effects of resistance training on central blood pressure in obese young men.Journal of human hypertension.2014, **28**(3):157-164. doi:10.1038/jhh.2013.81
7. Kon M, Ohiwa N, Honda A et al.Effects of systemic hypoxia on human muscular adaptations to resistance exercise training.Physiological reports.2014, **2**(6). doi:10.14814/phy2.12033
8. Farzanegi P, Zamani M, Khalili A et al.Effects of upper- and lower-extremity resistance training on serum vascular endothelial growth factor, myostatin, endostatin and follistatin levels in sedentary male students.Science & Sports.2021, **36**(2):139.e131-139.e136. doi:https://doi.org/10.1016/j.scispo.2020.02.013
9. Zhao Y, Lin A, Jiao L.Eight weeks of resistance training with blood flow restriction improve cardiac function and vascular endothelial function in healthy young Asian males.International health.2021, **13**(5):471-479. doi:10.1093/inthealth/ihaa089
10. DuttaRoy S, Nilsson J, Hammarsten O et al.High frequency home-based exercise decreases levels of vascular endothelial growth factor in patients with stable angina pectoris.European journal of preventive cardiology.2015, **22**(5):575-581. doi:10.1177/2047487314529349
11. Shimizu R, Hotta K, Yamamoto S et al.Low-intensity resistance training with blood flow restriction improves vascular endothelial function and peripheral blood circulation in healthy elderly people.European journal of applied physiology.2016, **116**(4):749-757. doi:10.1007/s00421-016-3328-8
12. Marston KJ, Brown BM, Rainey-Smith SR et al.Twelve weeks of resistance training does not influence peripheral levels of neurotrophic growth factors or homocysteine in healthy adults: a randomized-controlled trial.European journal of applied physiology.2019, **119**(10):2167-2176. doi:10.1007/s00421-019-04202-w
13. Beijer Å, Rosenberger A, Bölck B et al.Whole-body vibrations do not elevate the angiogenic stimulus when applied during resistance exercise.PloS one.2013, **8**(11):e80143. doi:10.1371/journal.pone.0080143
14. Pedrinolla A, Venturelli M, Fonte C et al.Exercise training improves vascular function in patients with Alzheimer's disease.European journal of applied physiology.2020, **120**(10):2233-2245. doi:10.1007/s00421-020-04447-w
15. Tsai CL, Pai MC, Ukropec J et al.Distinctive Effects of Aerobic and Resistance Exercise Modes on Neurocognitive and Biochemical Changes in Individuals with Mild Cognitive Impairment.Current Alzheimer research.2019, **16**(4):316-332. doi:10.2174/1567205016666190228125429
16. Cho SY, Roh HT.Taekwondo Enhances Cognitive Function as a Result of Increased Neurotrophic Growth Factors in Elderly Women.International journal of environmental research and public health.2019, **16**(6). doi:10.3390/ijerph16060962
17. Cho SY, So WY, Roh HT.The Effects of Taekwondo Training on Peripheral Neuroplasticity-Related Growth Factors, Cerebral Blood Flow Velocity, and Cognitive Functions in Healthy Children: A Randomized Controlled Trial.International journal of environmental research and public health.2017, **14**(5). doi:10.3390/ijerph14050454
18. Erbs S, Höllriegel R, Linke A et al.Exercise training in patients with advanced chronic heart failure (NYHA IIIb) promotes restoration of peripheral vasomotor function, induction of endogenous regeneration, and improvement of left ventricular function.Circulation Heart

failure.2010, **3**(4):486-494. doi:10.1161/circheartfailure.109.868992
19. Huffman KM, Slentz CA, Bales CW et al.Relationships between adipose tissue and cytokine responses to a randomized controlled exercise training intervention.Metabolism: clinical and experimental.2008, **57**(4):577-583. doi:10.1016/j.metabol.2007.11.023
20. Jiao X, Liu M, Li R et al.Helpful to Live Healthier? Intermittent Hypoxic/Ischemic Training Benefits Vascular Homeostasis and Lipid Metabolism with Activating SIRT1 Pathways in Overweight/Obese Individuals.Obesity facts.2024, **17**(2):131-144. doi:10.1159/000536093
21. Krogh J, Rostrup E, Thomsen C et al.The effect of exercise on hippocampal volume and neurotrophines in patients with major depression--a randomized clinical trial.Journal of affective disorders.2014, **165**:24-30. doi:10.1016/j.jad.2014.04.041
22. Lin S, Chen Y, Li Y et al.Physical ischaemia induced by isometric exercise facilitated collateral development in the remote ischaemic myocardium of humans.Clinical science (London, England : 1979).2014, **127**(10):581-588. doi:10.1042/cs20130618
23. Liu X, Zhu H, Peng Y et al.Twenty-Four week Taichi training improves pulmonary diffusion capacity and glycemic control in patients with Type 2 diabetes mellitus.PloS one.2024, **19**(4):e0299495. doi:10.1371/journal.pone.0299495
24. Maass A, Düzel S, Brigadski T et al.Relationships of peripheral IGF-1, VEGF and BDNF levels to exercise-related changes in memory, hippocampal perfusion and volumes in older adults.NeuroImage.2016, **131**:142-154. doi:10.1016/j.neuroimage.2015.10.084
25. Mai K, Klug L, Rakova N et al.Hypoxia and exercise interactions on skeletal muscle insulin sensitivity in obese subjects with metabolic syndrome: results of a randomized controlled trial.International journal of obesity (2005).2020, **44**(5):1119-1128. doi:10.1038/s41366-019-0504-z
26. Mortensen SP, Winding KM, Iepsen UW et al.The effect of two exercise modalities on skeletal muscle capillary ultrastructure in individuals with type 2 diabetes.Scandinavian journal of medicine & science in sports.2019, **29**(3):360-368. doi:10.1111/sms.13348
27. Soke F, Kocer B, Fidan I et al.Effects of task-oriented training combined with aerobic training on serum BDNF, GDNF, IGF-1, VEGF, TNF-α, and IL-1β levels in people with Parkinson's disease: A randomized controlled study.Experimental gerontology.2021, **150**:111384. doi:10.1016/j.exger.2021.111384
28. Taha M.Effect of High Intensity Interval Training on Endothelial Function in Postmenopausal Hypertensive Patients: Randomized Controlled Trial.International Journal of Physiotherapy.2016, **3**(1). doi:10.15621/ijphy/2016/v3i1/88908
